# *In silico* structural and functional prediction of African swine fever virus protein-B263R reveals features of a TATA-binding protein

**DOI:** 10.7717/peerj.4396

**Published:** 2018-02-22

**Authors:** Dickson Kinyanyi, George Obiero, George F.O. Obiero, Peris Amwayi, Stephen Mwaniki, Mark Wamalwa

**Affiliations:** 1 Department of Biochemistry and Biotechnology, Technical University of Kenya, Nairobi, Kenya; 2 Center for Biotechnology and Bioinformatics, University Of Nairobi, Nairobi, Kenya; 3 Department of Biochemistry and Biotechnology, Kenyatta University, Ruiru, Kenya

**Keywords:** African swine fever virus, TATA-binding protein, BA71V pB263R, *In silico* characterization, I-TASSER, Sequence homology search, African swine fever transcription factor

## Abstract

African swine fever virus (ASFV) is the etiological agent of ASF, a fatal hemorrhagic fever that affects domestic pigs. There is currently no vaccine against ASFV, making it a significant threat to the pork industry. The ASFV genome sequence has been published; however, about half of ASFV open reading frames have not been characterized in terms of their structure and function despite being essential for our understanding of ASFV pathogenicity. The present study reports the three-dimensional structure and function of uncharacterized protein, pB263R (NP_042780.1), an open reading frame found in all ASFV strains. Sequence-based profiling and hidden Markov model search methods were used to identify remote pB263R homologs. Iterative Threading ASSEmbly Refinement (I-TASSER) was used to model the three-dimensional structure of pB263R. The posterior probability of fold family assignment was calculated using TM-fold, and biological function was assigned using TM-site, RaptorXBinding, Gene Ontology, and TM-align. Our results suggests that pB263R has the features of a TATA-binding protein and is thus likely to be involved in viral gene transcription.

## Introduction

African swine fever virus (ASFV) is a large enveloped double-stranded DNA virus and the sole member of the family Asfarviridae (Dixon et al., 2004), which belongs to the nucleo-cytoplasmic large DNA virus (NCLDV) superfamily, an apparently monophyletic class of viruses with eukaryotic hosts. NCLDVs partially replicate in the cytoplasm, with varying degrees of dependence on the host nucleus (Dixon et al., 2013); members of the superfamily have been classified to belong to the proposed new order named Megavirales, some include, Poxviridae, Iridoviridae, Mimiviridae, Phycodnaviridae, Marseilleviridae, and Ascoviridae (Colson et al., 2012; Colson et al., 2013). The natural hosts of ASFVs include bush pigs, warthogs, and argasid ticks of the genus *Ornithodoros* (Carrillo et al., 1994; Rodríguez & Salas, 2013) in these hosts, infection by the virus is not fatal. ASF in domestic pigs is characterized by lethal hemorrhagic fever and a mortality rate of up to 100%. The most effective control strategies are quarantine and slaughter of infected and potentially infected animals (Zsak et al., 1998; Afonso et al., 2004). There is currently no vaccine against ASFV (Donnell et al., 2015; Reis et al., 2016), making it a major threat to the global pork industry.

Complete ASFV genome sequences contain between 151 and 167 open reading frames closely spaced along both strands of the viral genome (Dixon et al., 2013; Rodríguez & Salas, 2013). About half of open reading frames lack a known or predicted function (Yáñez et al., 1995; Chapman et al., 2008; Rodríguez & Salas, 2013); most gene products are classified as hypothetical proteins or uncharacterized proteins (Dixon et al., 2013). Moreover, little is known about the molecular mechanisms controlling ASFV gene transcription (Rodríguez & Salas, 2013). Late promoters of ASFV gene sequences are enriched in A/T bases, with an average A/T content of 80% as compared to 61%–62% for the BA71V genome (Rodríguez & Salas, 2013). Comparative analysis of late promoter sequences from various ASFV genomes have revealed conservation of A/T-rich regions that bear similarity to TATA motifs at or near the transcription start site (García-Escudero & Viñuela, 2000; Rodríguez & Salas, 2013). Mutations and linker substitutions/deletions in these TATA-like sequences alter the transcriptional activity of late genes such as B646L, K78R, EP402R, and A137R (Rodríguez & Salas, 2013) as well as that of p11.5, p10, and p54 (García-Escudero & Viñuela, 2000). Although putative ASFV transcription factors have been identified including transcription initiation factor IIB-like factor (C315R) and transcription initiation factor IIS encoded by the I243Lgene (Iyer, Aravind & Koonin, 2001; Dixon et al., 2013), there are no reports of transcription factors in ASFV that bind TATA-like sequences (García-Escudero & Viñuela, 2000).

To this end, the present study investigated the three-dimensional (3D) structure and function of pB263R using an *in silico* approach. We used sequence-based methods to identify remote homologs of pB263R and model protein structure. Functional prediction methods were used to infer binding sites and ligands.

## Materials and Methods

### Sequence-based similarity search

The BA71V pB263R open reading frame (RefSeq: NP_042780.1) was obtained from UNIPROT (Magrane & UniProt Consortium, 2011; Wasmuth & Lima, 2016) (http://www.uniprot.org/uniprot/Q65175) and used for an initial search against the National Center for Biotechnology Information non-redundant database using PSI-BLAST (Altschul et al., 1997) default parameters. Since there were no experimentally solved template sequences with known function identified by PSI-BLAST, we used HHpred (Söding, Biegert & Lupas, 2005; Alva et al., 2016) (https://toolkit.tuebingen.mpg.de/hhpred)—a profile sequence search algorithm—to search for remote homologous protein families against pfam04jul16 (Bateman et al., 2002; Finn et al., 2016), SCOPe2.06 (Fox, Brenner & Chandonia, 2014), and PDBv3.3 (Bernstein et al., 1977) non-redundant databases with three maximum multiple sequence alignment generation iterations and an *E*-value cut-off of 0.0001.

### Structural modeling and classification of pB263R

Within I-TASSER (http://zhanglab.ccmb.med.umich.edu/I-TASSER/) (Roy, Kucukural & Zhang, 2010; Yang & Zhang, 2015), the top 10 protein templates independently identified by LOMETS (Wu & Zhang, 2007) were used to model the 3D structure of both full-length and truncated pB263R protein. For the full-length sequence, RaptorX (http://raptorx.uchicago.edu/StructurePrediction/predict/) (Peng & Xu, 2011; Källberg et al., 2012; Ma et al., 2013) was queried to analyze pB263R topology. We searched for structural analogs of pB263R model 1a using TM-align (Zhang & Skolnick, 2005; Roy, Yang & Zhang, 2012), and the best template was superimposed and visualized with ICM Browser (Raush et al., 2009) (V.3.8.5) (http://www.molsoft.com/icm_browser.html). The top 10 structural analogs of pB263R model 1a identified by TM-align were queried in TM-fold (Xu & Zhang, 2010) (http://zhanglab.ccmb.med.umich.edu/TM-fold/readme.html) to verify their posterior probabilities of fold family classification.

### Structural validation

Quality of the predicted structure was analyzed using structural validation algorithms ProSA (Wiederstein & Sippl, 2007) and Rampage (Lovell et al., 2003).

### Functional and ligand binding site annotation

The COACH (Yang, Roy & Zhang, 2013a; Yang, Roy & Zhang, 2013b) (http://zhanglab.ccmb.med.umich.edu/COACH/) bundled TM-site based on existing benchmarked studies was used to compare the structure of a sub-sequence from the first to last binding residues of the queried 3D structure against the Biolip database (Yang, Roy & Zhang, 2013a) (http://zhanglab.ccmb.med.umich.edu/BioLiP/database) to identify potential ligand-binding site residues in the predicted pB263R model 1a structure. Sequence-based pocket and ligand binding sites of pB263R were independently predicted using the RaptorX binding site prediction server (http://raptorx.uchicago.edu/BindingSite/) (Källberg et al., 2012). A multiple sequence alignment was performed for pB263R model 1a, and the 10 highest-ranked structural analogs of the predicted model were identified with the RaptorX structure alignment server at (http://raptorx.uchicago.edu/DeepAlign/submit/) and visualized using JalView v.2.10.1 (Waterhouse et al., 2009) to infer function and identify conserved residues. GO terms (Huntley et al., 2015) predicted with the I-TASSER server were used to infer the pB263R model 1a functional annotation.

### Phylogenetic analysis

Protein sequences of TBP, TBP-like factor, and transcription initiation factor II D subunits comprising the transcription factors predicted by HHPred as well as pB263R and TBPs from representative members of the Poxviridae, Iridoviridae, Mimiviridae, Phycodnaviridae, Marseilleviridae, and Ascoviridae families obtained from the UNIPROT database were aligned with MUSCLE (Edgar, Drive & Valley, 2004) using MEGA6 (Tamura et al., 2013). A pairwise distance matrix and a phylogenetic tree of the aligned sequences were generated by the maximum likelihood method. A phylogeny bootstrap test was carried out based on a preliminary neighbor-joining inferred tree; 1,000 bootstrap replicates were used to generate a final consensus tree.

## Results and Discussion

### pB263R sequence analysis

A search for ASFV pB263R (RefSeq: NP_042780.1) homologs against the National Center for Biotechnology Information non-redundant database using PSI-BLAST with a threshold of 0.005 reached convergence at the 5th iteration and failed to return functional homologs of the queried protein sequence. The identified sequences were putative proteins conserved among ASFV and faustovirus, another member of the NCLDV superfamily (Oliveira et al., 2017). The pB263R sequence was probed in HHpred (Söding, Biegert & Lupas, 2005; Alva et al., 2016), a more sensitive sequence-based algorithm for remote homolog detection. The results revealed that pB263R had 12%–15% sequence identity to TATA-binding domains of *Methanocadococcus jannaschii*, *Pyrococcus woesei*, *Saccharomyces cerevisiae*, *Sulfolobus acidocaldarius*, and *Encephalitozoon cuniculi*. *E* values were below the 1 × 10^−4^ cut-off level, indicating that they were significant and had a 100% probability of being true positive (Table 1). Based on the hidden Markov model, we did not expect good structural predictions for amino acid residues ∼1–44.

**Table 1:** pB263R homologs identified by HHpred.

Rank	PDB ID	Organism	Protein domain	Probability^a^	*E* value	Identity^b^	Query HMM region	Columns^c^
1	2z8uA	*M. jannaschii*	TBP	100%	7.6 × 10^−39^	15%	44–253	176
2	1aisA	*P. woesei*	TBP	100%	1.3 × 10^−38^	15%	40–251	178
3	1ytbA	*S. cerevisiae*	TBP	100%	2.1 × 10^−38^	15%	44–253	175
4	1mp9A	*S. acidocaldarius*	TBP	100%	3.7 × 10^−38^	12%	44–263	185
5	3eikA	*E. cuniculi*	TPB	100%	1.0 × 10^−37^	13%	34–253	185

**Notes.**

a Probability of the template being a true positive.

b Percent sequence identity between the template and query sequences.

c Number of aligned HMM-HMM match-match columns.

HMM hidden Markov model PDB ID Protein Data Bank identification TBP TATA-binding protein

**Figure 1:**
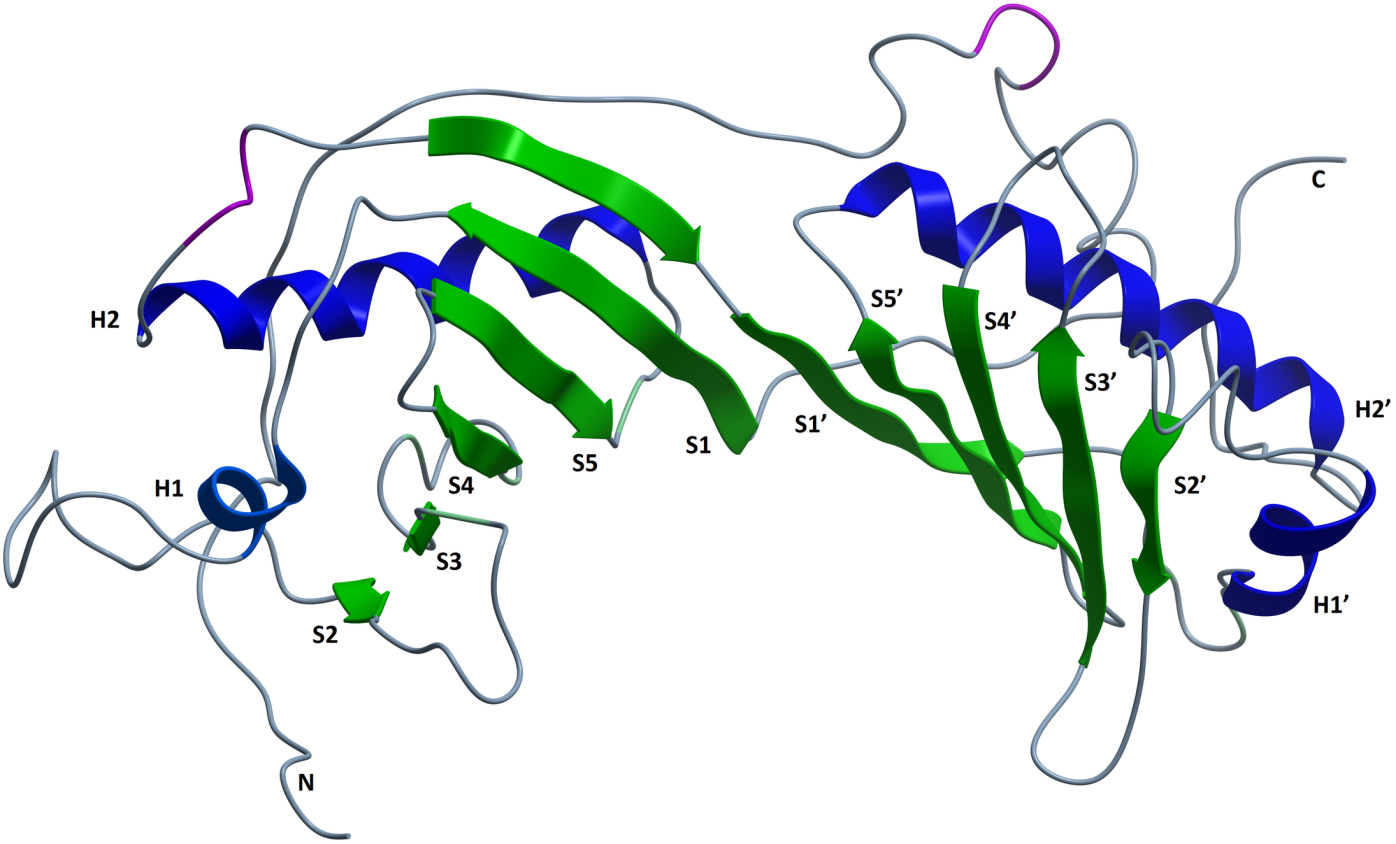
I-TASSER-modeled structure of the complete pB263R protein sequence (residues 1–263). A ribbon-style representation of the predicted 3D model (pB263R model 1a) is shown, with a TM score of 0.52 ± 0.15 and Cs of −1.61. The *β* strands and *α* helices form a pseudo-dyad structure consisting of two similar subdomains, each with five *β* strands (S1–S5 on subdomain 1 and S1′–S5′ on subdomain 2) and two *α* helices (H1/H2 and H1′/H2′). N and C indicate the N and C termini, respectively, of pB263R model 1a.

### Structural modeling, validation and classification of pB263R

**Figure 2:**
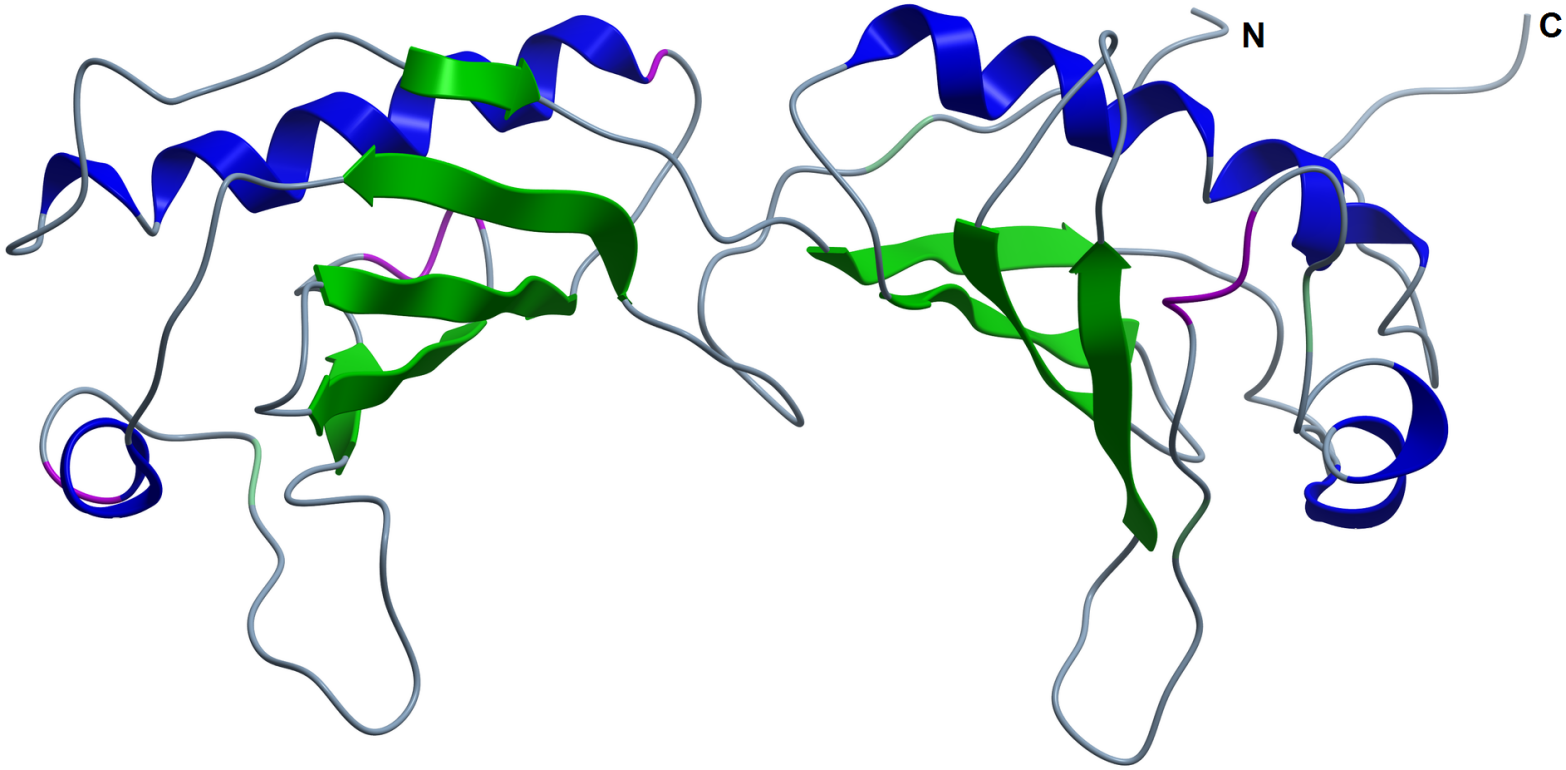
Model of pB263R residues 45 (A1) to 263 (D219) predicted with I-TASSER. The structure was consistent with typical TBP topology but with an asymmetric number of anti-parallel *β* sheets. The structure had a high TM score (0.57) and Cs (−1.15), indicating that the loop from residues 1 to 44 contained a possible species-specific intrinsically disordered region.

**Figure 3:**
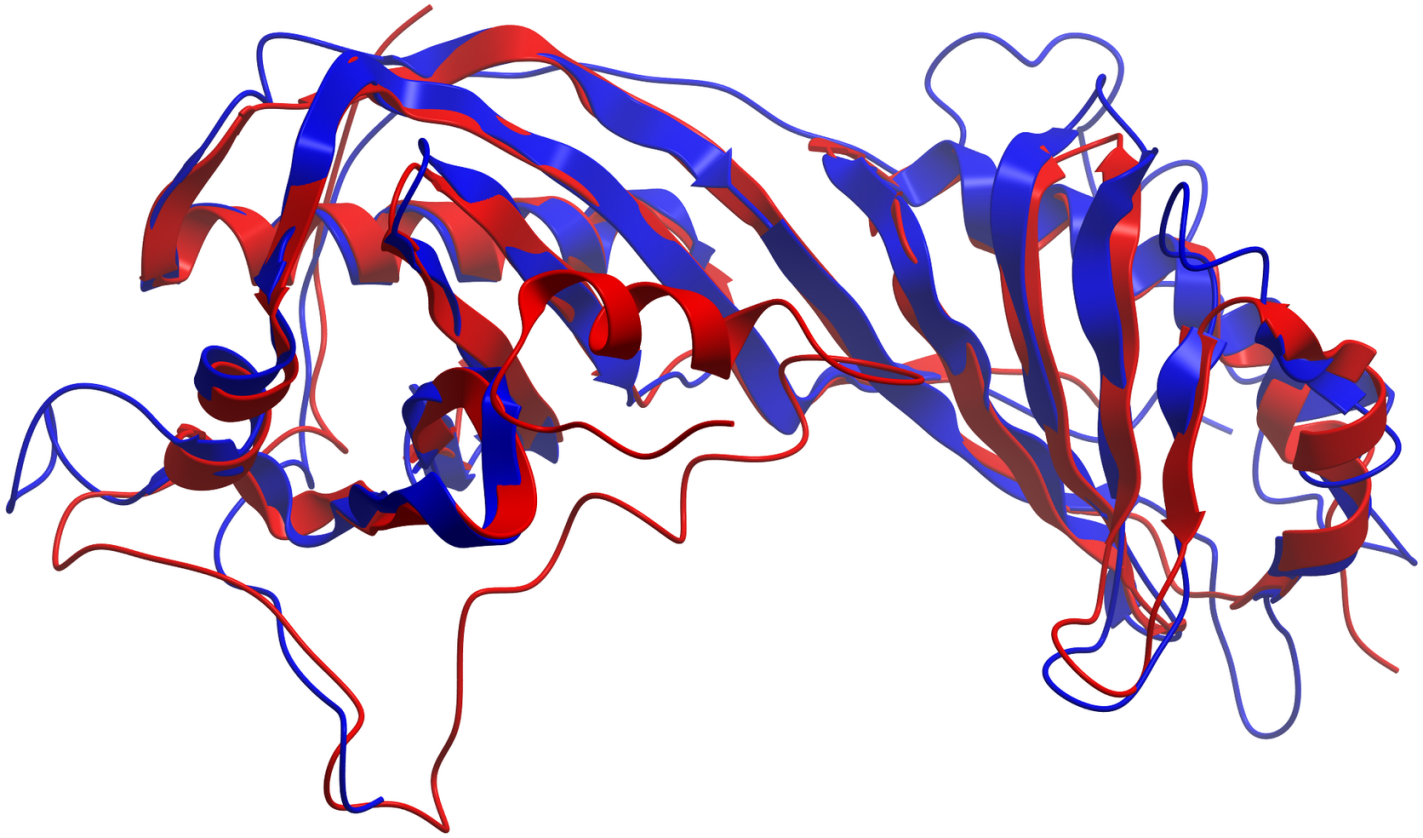
TM-align structural superposition of pB263R model 1a (blue ribbon) and experimentally solved 4b0aA. The TM score was 0.711. The figure was generated using the molsoft icm browser (http://www.molsoft.com/icm_browser.html).

Iterative Threading ASSEmbly Refinement (I-TASSER)-based Local Meta-Threading Server (LOMETS) was used to model the pB263R 3D structure, with the top 6 of 10 independently identified threading templates having a normalized Z score >1, which indicated a good alignment. The predicted 3D structure was thus expected to be highly accurate (Roy, Kucukural & Zhang, 2010). There was a general consensus among independent algorithms in LOMETS; all top-scoring experimentally solved proteins with normalized Z scores >1 were TATA-binding proteins (TBPs) (Table S1 and Data S1). The first of five models (pB263R model 1a) (Fig. 1) predicted as a full-length simulation from the pB263R sequence was obtained as a 3D coordinate file from the I-TASSER online server (Roy, Kucukural & Zhang, 2010). pB263R model 1a had a statistically significant template modeling (TM) score of 0.52. The TM score reflects the topological similarity of protein structure pairs and has a value in the range of [0,1], with a higher score indicating a better structural match. Thus, a TM score <0.17 represents a randomly selected protein pair whereas a score >0.5 corresponds to protein pairs of similar fold, confirming a correct topology (Xu & Zhang, 2010; Yang et al., 2015). The confidence score (Cs) of pB263R model 1a (Fig. 1) was −1.61 due to non-matching regions in an extended N-terminal opening loop from sequence 1–44. Removing the intrinsically disordered and non-conserved N terminus and querying the I-TASSER server with the pB263R sequence from residues 45 to 263 improved the TM score to 0.57 with a Cs of −1.15, which was within the limits of the threshold set for statistical significance (Zhang, 2008). The structure predicted for this residue range (45–263) was a TBP-like domain (Fig. 2 and Data S2). Predictions by RaptorX (Figure S2) and HHpred (Table 1) suggested the presence of a long N-terminal coil (from residues ∼1–44). In the full sequence-based structure of pB263R with a species-specific N terminus, most structural alignments of the C-terminal domain (CTD) coincided with the core region of the TBP fold; moreover, the predicted pB263R model 1a had similar fold topology to 4b0aA (Anandapadamanaban et al., 2013), a TBP (Fig. 3). The pB263R model 1a structure (Fig. 1) predicted by I-TASSER had a typical pseudo-dyad symmetric *α*∕*β* saddle-like structure with bifurcating stirrups. Each subdomain of the CTD was populated with *α* helices (H1/H2 and H1′/H2′) and *β* strands (S1–S5 and S1′–S5′). The latter formed the curved underside of the structure binding to the minor groove of the TATA element in the DNA sequence (Juo et al., 1996; Patikoglou et al., 1999).

**Figure 4:**
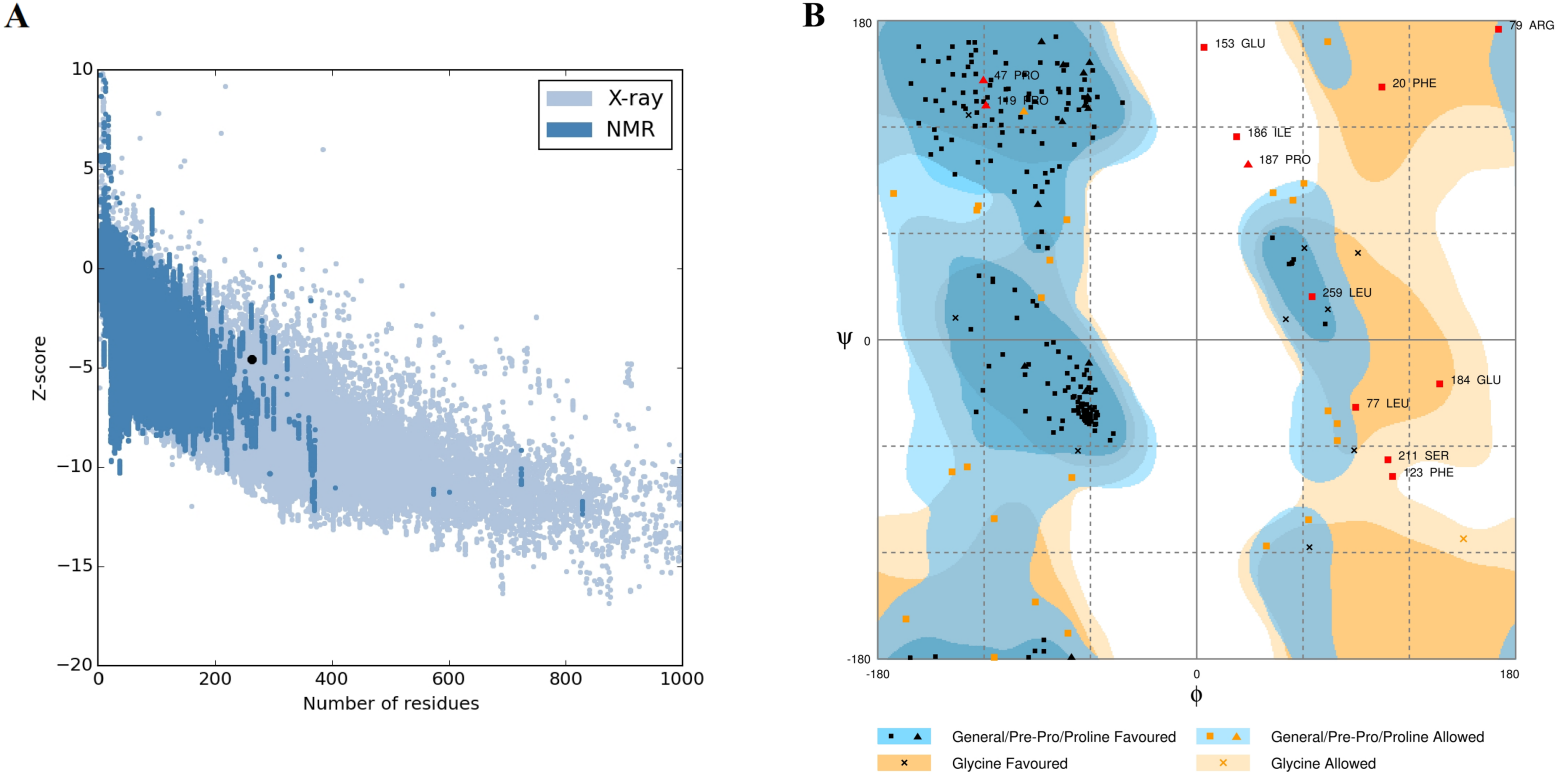
pB263R in silico predicted structure validation. The predicted structure was validated using ProSA Web and rampage. (A) ProSA Web plot showing *Z*-score of the predicted structure −4.65 of pB263R (black dot) relative to *Z*-scores of similar sized protein structures solved using NMR and X-ray crystallography. (B) Ramachandran plot obtained from Rampage showing 85.4% of residues lie in the most favoured region, 9.6% of residues in allowed regions and 4.6% in the outlier region.

The full sequence predicted structure was additionally assessed by structure validation algorithms Rampage and ProSA web. ProSA compares the *Z*-Scores of the predicted structures against protein structures of the same size obtained by NMR and X-ray crystallography. The predicted model obtained a *Z*-Score of −4.56 which falls within the *Z*-score of similar sized protein structures (Fig. 4A). A ramachandran plot obtained from Rampage, an algorithm that validates the general stereochemical quality of a protein structure in a plot showing the phi and psi angles for each residue of a protein, indicated the predicted model to have acceptable 85.8% in favoured region and 9.6% in the allowed region (Fig. 4B).

Structural PDB analogs of pB263R model 1a were identified with TM-align (Zhang & Skolnick, 2005), a protein structure alignment algorithm, whereas posterior probabilities of family fold classification for identified PDB analogs with the highest degree of similarity to pB263R model 1a (Table 2) were determined with TM-fold (Xu & Zhang, 2010). Posterior probability is the probability that two proteins have the same fold family assignment in either Structural Classification of Proteins (SCOPe) (Fox, Brenner & Chandonia, 2014) or Class, Architecture, Topology, and Homology (CATH) (Sillitoe et al., 2015) structural classification databases for a given TM score (Xu & Zhang, 2010). For pB263R model 1a, the top eight of 10 experimentally solved TBP structures identified by TM-align had >97.5% posterior probability of sharing a similar fold with pB263R model 1a in both SCOPe and CATH (Table 2). The highest ranked structural analog 4b0aA had a TM score of 0.711; the TM-fold tabulated (non-manual) posterior probability findings revealed that the two structures (i.e., 4b0aA and pB263R model 1a) belonged to the same fold family with posterior probabilities of 98.32% in CATH and 98.81% in SCOPe databases. However, the experimentally solved 4b0aA did not have a manual classification in SCOPe 2.06 (Fox, Brenner & Chandonia, 2014) (Table 2); therefore, the second ranked structure 1vokA was considered for fold family classification based on the fact that it had prior classification in both SCOPe 2.06 and CATH. Classification of pB263R model 1a using 1vokA (Table 2) yielded posterior probabilities of 99.18% in SCOPe and 99.66% in CATH of the two structures belonging to the same family (CATH Code: 3.30.310.10, Class, alpha beta, Architecture, Two-layer sandwich, Topology, TBP, Homologous superfamily, TATA-binding protein). In SCOPe 2.06, 1vok shared a similar classification of d.129.1.1 (Class d: of *α* and *β* proteins, Fold of TBP-like fold d.129, Superfamily d.129.1 of TBP-like and d.129.1.1, Family of TBP,C-terminal domain). pB263R model 1a was therefore structurally classified as a TBP. Additionally, TM-fold non-manually classified 4b0aA as belonging to the same fold family as 1vokA, with posterior probabilities of 99.70% in SCOPe and 99.19% in CATH, yielding a TM score of 0.9142. However, function prediction studies are necessary to determine whether pB263R model 1a is a TBP since proteins with similar folds can have distinct functions (Skolnick et al., 2015).

**Table 2:** Top 10 experimentally solved structural analogs of pB263R model 1a identified by TM-align.

Rank^a^	PDB hit	**SCOPe classification**	Non-normalized TM score	RMSD^b^	IDEN^c^	Coverage^d^	Posterior probability in SCOPe^e^	Posterior probability in CATH^f^
1	4b0aA	Not manually classified in SCOP 2.06	0.711	1.49	0.162	0.715	0.9881	0.9832
2	1vokA	d.129.1.1	0.670	1.64	0.135	0.696	0.9918	0.9966
3	1jfiC	d.129.1.1	0.664	1.45	0.115	0.684	0.9958	0.9954
4	1mp9A	d.129.1.1	0.657	2.09	0.125	0.677	0.9958	0.9910
5	1nh2A	d.129.1.1	0.656	1.31	0.167	0.684	0.9920	0.9914
6	3eikA	d.129.1.0	0.645	1.43	0.146	0.688	0.9920	0.9369
7	1pczA	d.129.1.1	0.643	1.88	0.158	0.665	0.9918	0.9964
8	2z8uB	d.129.1.1	0.618	1.68	0.161	0.677	0.9919	0.9098
9	2glsL	d.15.9.1	0.486	5.48	0.061	0.681	0.2902	0.1236
10	3ng0A	d.15.9.0	0.481	5.66	0.057	0.707	0.2736	0.1150

**Notes.**

a Rank of PDB structures based on TM score of the structural alignment between the pB263R model 1a and known structures in the PDB library.

b Root mean square deviation between residues structurally aligned by TM-align.

c Percent sequence invariability in structurally identical regions.

d Alignment coverage by TM-align, equivalent to the number of structurally aligned residues divided by the length of the queried protein sequence.

e Probability of two proteins structures being in the same fold family in SCOPe.

f Probability of two proteins structures being in the same fold family in CATH.

CATH Class, Architecture, Topology, and Homology PDB Protein Data Bank SCOPe Structural Classification of Proteins

**Table 3:** Top five predicted binding site residues for pB263R as determined by TM-site.

Rank	CSt^a^	Cluster size^b^	Representative template^c^	TATA box sequence	Ligands^d^	Predicted binding site residues in pB263R model 1a
1	0.39	32	1qn4A_BS01_NUC	TGCC[CATTTATA]GC (TATAAATG)	Nucleic acid (32)	N52, N54, F90, N91, K108, F110, T113, E115, Q117, I157, Q158, N160, E200, D201, S205, F207, R217, N219, L229, and N232
2	0.37	27	1qnbA_BS02_NUC	TGCC[CATTTATA]GC (TATAAATG)	Nucleic acid (27)	K49, A50, N52, C89, F90, E95, I98, M104, K106, K108, P119, G120, N160, D201, S202, N219, F221, K223, K225, and N227
3	0.19	3	5fmfQ_BS02_NUC	C[CTTTTATA]G (TATAAAAG)	Nucleic acid (3)	K49, A50, T88, C89, F90, N91, A93, E95, S97, M104, K106, K108, F110, P119, G120, I122, D201, S202, F221, K223, and K225
4	0.16	1	5fz5O_BS01_NUC	C[CTTTTATA]G (TATAAAAG)	Nucleic acid (1)	N52, N54, L60, F90, F110, S112, E115, Q117, Q158, D201, and L229
5	0.16	1	5fz5O_BS02_NUC	T[ATTATATA]CA (TATATAAT)	Nucleic acid (1)	K49, N50, N52, C89, F90, E95, L99, K106, K108, P119, and S202

**Notes.**

a Confidence score of the TM-site prediction (rang: 0–1).

b Total number of templates in a cluster.

c Single complex structure with the most representative ligand in the cluster.

d Name of identified ligand.

**Figure 5:**
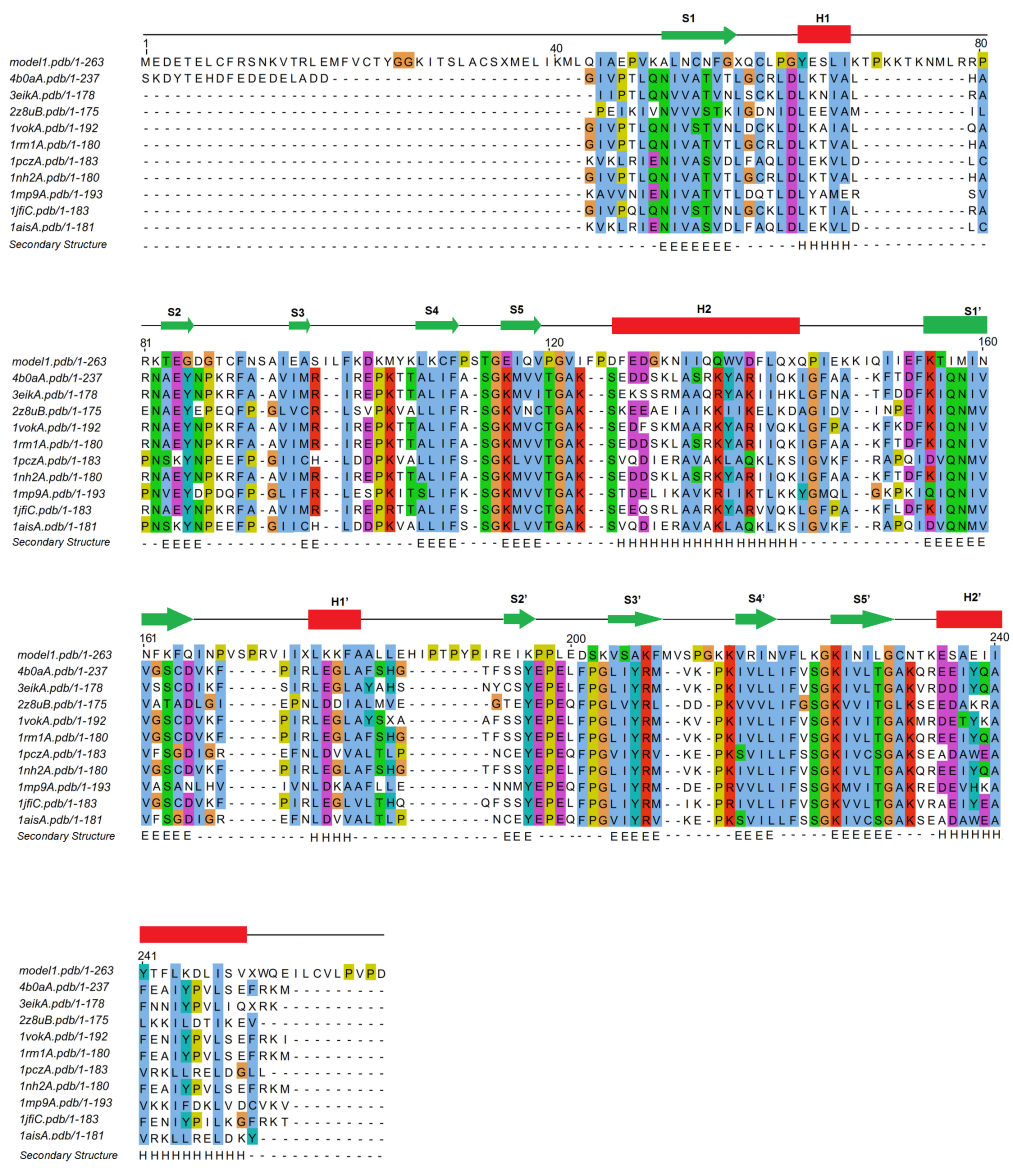
Multiple sequence alignment of TBP sequences of the top 10 closest TBP structural homologs of pB263R. 4b0aA, *S. cerevisiae* TBP transcription initiation factor II D subunit 1; 3eikA, *E. cuniculi* TBP; 2z8uB, *M. jannaschii* TBP; 1vokA, *A. thaliana* TBP; 1rm1A, *S. cerevisiae* TBP; 1pczA, *P. woesei* TBP; 1nh2A, *S. cerevisiae* TBP CTD; 1mp9A, *S. acidocaldarius* TBP; 1jfiC, Homo sapiens TBP; 1aisA, *P. woesei* TBP. F90, F110 and F223 amino acid residues are indicated to be highly conserved among TBP.

**Figure 6:**
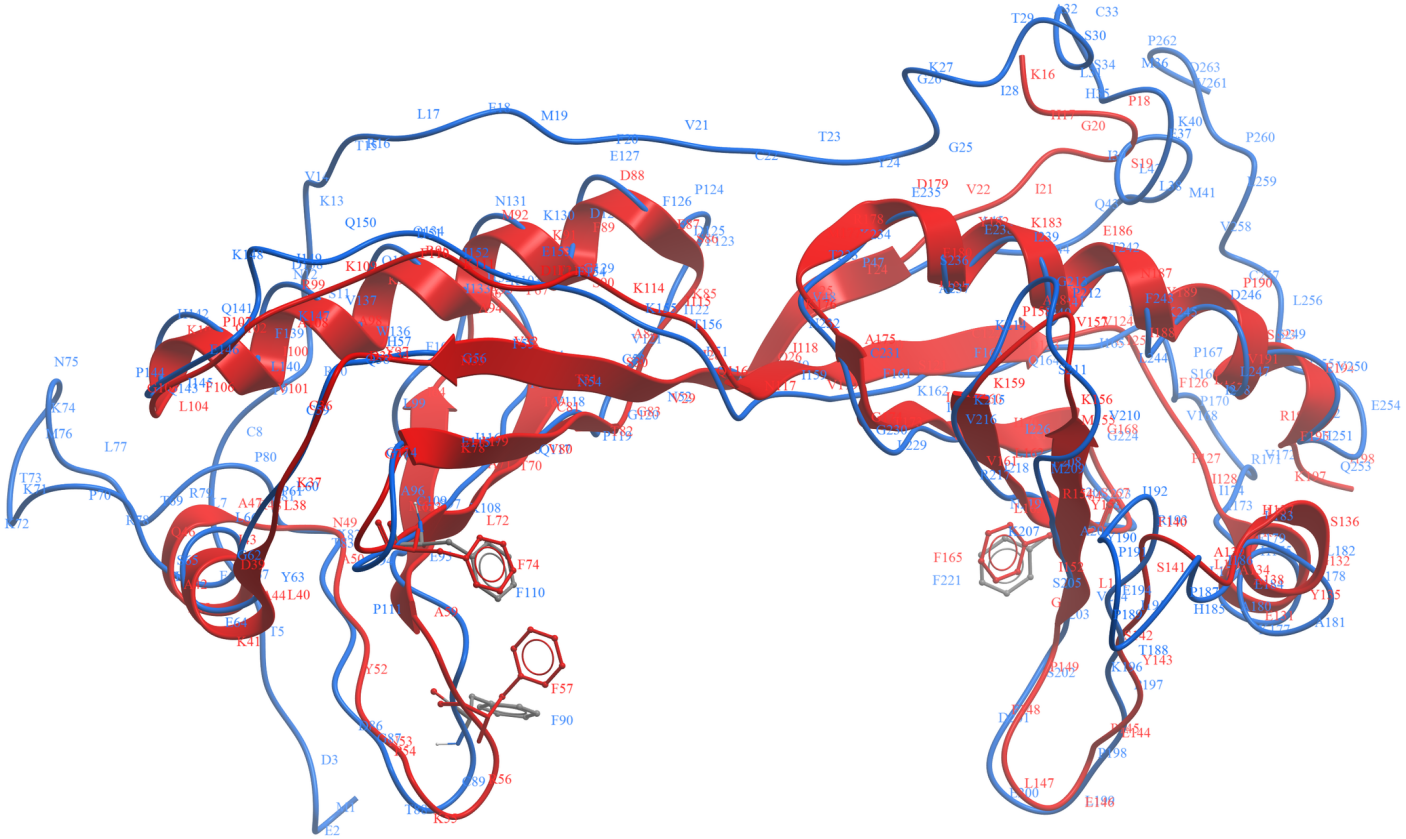
Structural superimposition of pB263R model 1a (blue coil) and experimentally solved 1qn4A (red ribbon). The predicted conserved TATA box element includes intercalating residues F90, F110, and F221 of pB263R model 1a on the underside of concave *β* sheets.

### Prediction of pB263R model 1a biological function and ligand binding site

TM-site–a function prediction algorithm–identified the structures of 1qn4A and 1qnbA (Patikoglou et al., 1999), two TBP isoforms in *Arabidopsis thaliana*, as single complex structures most representative of identified ligand clusters with TM-site Confidence-scores (CSt) of 0.39 (1qn4A) and 0.37 (1qnbA). These scores were significantly higher than the default cut-off value for good ligand binding site prediction (0.35) from a previously benchmarked training set in which a CSt > 0.35 was associated with average false positive and false negative rates below 0.16 and 0.13, respectively (Yang, Roy & Zhang, 2013a), implying shared binding sites with pB263R model 1a. Ligand binding sites for both structures were transferred to pB263R model 1a as putative binding sites, with 20 and 21 binding site residues being mapped from 1qn4A and 1qnbA, respectively (Table 3). The ligand binding site predictions from the modeled structures and multiple sequence alignment (Fig. 5) revealed three conserved phenylalanine residues (F90, F110, and F221) on the curved underside of the C-terminal domain of pB263R model 1a. The positions of these three residues corresponded to those involved in TATA element kinking (Figs. 5 and 6). Our study did not provide any direct insight into the precise bending and kinking mechanisms in pB263R model 1a and other TBPs containing three conserved phenylalanine residues—for example, those of *Perkinsus marinus*, *Cryptosporidium parvum*, and *Toxoplasma gondii*—that typically lack TBP-associated factor 1/Mot1p regulators of TBP function (Koster, Snel & Timmers, 2015). However, based on TBP association with TATA elements, this is not the first report of a TBP with three phenylalanine residues associating with a TATA-like element and inducing transcriptional activation. Promoter recognition studies in *Plasmodium falciparum*, which, similar to ASFV, has an AT-rich genome and contains three conserved phenylalanine residues in its TBP, found that *Plasmodium falciparum* TBP (PfTBP) can recognize not only typical TATAA box sequences in the knob-associated histidine rich protein promoter, but also close variants of TATA elements in other promoters such as TGTAA in the glycophorin-binding protein-130 promoter region (Ruvalcaba-salazar et al., 2005). In addition to binding to conserved conserved TATA elements, TBPs with three intercalating phenylalanine residues may exhibit functional diversity in binding TATA variants. Consistent with these TATA binding observations, the statistically significant prediction of TM-site was pB263R model 1a bound to TATAAATG. TATAAATG met the functional definition of a TATA box sequence (Juo et al., 1996; Patikoglou et al., 1999). Similar TATA sequences both in composition and function have been detected in 5′-flanking sequences of late ASFV genes (García-Escudero & Viñuela, 2000; Rodríguez & Salas, 2013). A comparison of the composition of a representative conserved ASFV sequence (TATATA) observed in 5′-flanking sequences of late ASFV genes (García-Escudero & Viñuela, 2000; Rodríguez & Salas, 2013), *P. falciparum* (TATAA) consensus TATA box located 81 base pairs upstream of the transcription start site in the knob-associated histidine rich protein promoter region (Ruvalcaba-salazar et al., 2005), and *A. thaliana* 1qn4A (TATAAATG) ligand sequence showed conservation of bases critical for TATA binding in the upstream half of TATA box elements (Juo et al., 1996). Both *P. falciparum* TBP (Ruvalcaba-salazar et al., 2005) and *A. thaliana* (Patikoglou et al., 1999) TBP have been shown to associate with conserved consensus TATA box sequences and thereby induce transcriptional initiation. It is therefore highly likely that the predicted pB263R model 1a binds to these conserved ASFV TATA sequences. Moreover, the finding that the replacement, by GCGC, of the equivalent TATA sequence on the late genes K78R, EP402R and A137R was deleterious for transcription activity , further suggests that the TATA sequence could be a motif for late promoter function (García-Escudero & Viñuela, 2000; Rodríguez & Salas, 2013). Independently, the RaptorX binding site recognition server reproducibly predicted eight pockets for pB263R that exceeded the threshold multiplicity value of 40 (http://raptorx.uchicago.edu/documentation/#goto2) (Table 4), indicating that they occurred frequently in the protein sequence. The bases identified by the RaptorX binding site recognition server (TTAATAAG) corresponded to Adenine/Thymine elements and a Guanine in the eighth pocket; this was somewhat similar to a functional TATA element, reinforcing the possibilitythat pB263R binds to TATA elements in ASFV. Additionally, positively charged lysine residues and arginine that can bind to the negatively charged phosphate backbone of DNA (Nikolov et al., 1995)—i.e., K49, K106, K108, K203, K215, K162, R217, K223, and K225–were also be observed from the putative transfer of residues from TM-site and RaptorX binding site recognition server (Tables 3 and 4). Multiple sequence alignment (Fig. 5) also showed conservation of pB263R glycine residues (Gly114, Gly120, Gly224, and Gly230) in both CTD subdomains. These glycine residues are conserved in most eukaryotic TBPs and are required for a 3D structure that accommodates a short turn between critical *β* strands (S4, S4′ and S5, S5′) (Table 3). Gly120 and Gly230 have also been shown to make water-mediated contacts via carbonyl oxygen atoms (Nikolov et al., 1995). Proline residues (P111, P119, P124, P144, P167, P170, P189, P191, P198, and P212) were also observed (Figure S1) in the turns of *α* helices and *β* strands within the predicted TBP topology, and most valine residues comprising the hydrophobic core were replaced with asparagine (N) residues that were presumably involved in hydrogen bond formation with TATA bases (V → N227, L → N219, V → N160, V → N52 (S/T) → N54, and V → Q117).

**Table 4:** Binding pockets predicted by RaptorX binding based on pB263R sequence.

Pocket	Pocket multiplicity^a^	Ligand^b^	Binding residue
1	146	DT	D201, K203, S205, N219, and L229
2	141	DT	C89, F90, S97, K106, K108, and Q117
3	110	DA	N52, N54, F110, E115, Q117, and I157
4	104	DA	N52, I157, M158, N160, R217, L229, and G230
5	96	DT	K49, A50, N52, L99, K106, P119, G120, and N160
6	94	DA	K49, N160, K162, F221, K225, and N227
7	94	DA	D201, S202, F221, K223, and K225
8	82	DG	F90, F110, T113, and E115

**Notes.**

a Frequency with which the predicted pocket was found in the query protein template structure.

b DA, 2′-deoxyadenosine-5′-monophosphate; DG, 2′-deoxyguanosine-5′-monophosphate; DT, thymidine-5′-monophosphate.

**Figure 7:**
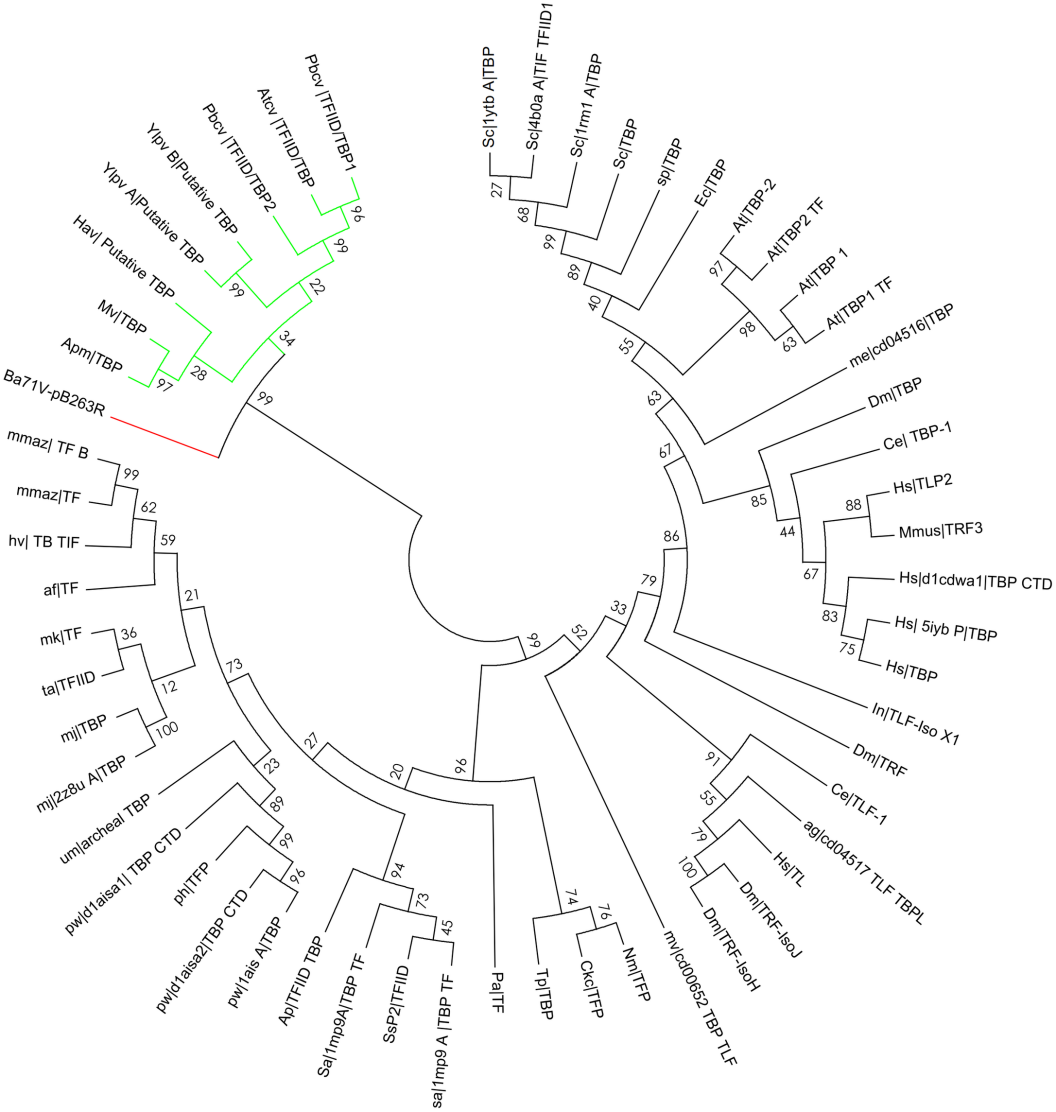
Bootstrap phylogenetic tree of pB263R with archaeal, eukaryotic, and prokaryotic TBPs and related factors. Light green indicates NCLDV TBPs while red indicates BA71V pB263r. Values at nodes indicate maximum likelihood bootstrap percentages. pB263r (red) and NCDLVs TBPs (light green) suggesting a closer evolutionary relatedness. Phylogenetic analyses were performed with MEGA6.

Based on a previously benchmarked dataset with a Gene Ontology (GO) score cut-off of 0.5, 85.1% of molecular functions, 76.9% of biological processes, and 74.6% of cellular locations can be correctly assigned (Roy, Kucukural & Zhang, 2010). The consensus predicted function for pB263R based on GO terms (Table S2 and Data S1) was a DNA-binding protein (GO: 003677; GO score = 0.78). The biological processes suggested were transcription initiation from RNA polymerase II promoter (GO: 0006367; GO score = 0.78) and any process modulating the frequency or extent of transcription based on a DNA template (GO: 0006355; GO score = 0.78). The cellular component suggested a DNA-directed RNA polymerase II holoenzyme (GO: 0016591, GO score: = 0.52) (Dimmer et al., 2008; Huntley et al., 2015). These findings further suggest that pB263R binds to promoters via selective and non-covalent interaction with DNA sequences and modulates RNA polymerase assembly at these promoters.

### Evolutionary relationship of pB263R to other TBPs

To investigate the evolutionary relatedness of pB263R to other TBPs, we compared the pB263R sequence of the BA71V strain with those of other NCLDV members and with TBP sequences from *S. cerevisiae*, *H. sapiens*, and *A. thaliana* possessing a CTD and TBP transcription initiation factor II D activity, as predicted by HHPred. Sequences determined by a pairwise distance matrix to have non-conserved n/c scores were omitted from the phylogenetic analysis. Putative TBPs of NCLDVs members (Yellow Lake phycodnavirus, *Acanthamoeba polyphaga* mimivirus, *Paramecium bursaria* Chlorella virus, *Acanthocystis turfacea* Chlorella virus, Heterosigma akashiwo virus, Megavirus, and *Acanthamoeba castellanii* mimivirus) and of pB263R isolate from BA71V showed moderate similarity and formed a single clade in the inferred maximum likelihood bootstrapped phylogenetic tree (Table S3 and Fig. 7). Proteins are classified based on similarities in function, structural design, and organism type (Thomas & Chiang, 2006). Our phylogenetic analysis suggests that pB263R is a TBP protein with homologs in other NCLDVs.

## Conclusion

In this study, we predict the structure and function of an uncharacterized protein B263R from an array of independent algorithms employing sequence, structure and classification based methods. We report the first TBP prediction in ASFV and bridge the proposed gap of elucidating the function of the TATA element in ASFV late transcription as well as identifying the possible transcriptional factors which could interact with it (García-Escudero & Viñuela, 2000), B263R being one such transcription factor.

##  Supplemental Information

10.7717/peerj.4396/supp-1 Table S1 Top ten analogs identified by LOMETS for threading alignment. (A) Iden1 is the percentage sequence identity of the templates in the threading aligned region with the query sequence. (B) Iden2 is the percentage sequence identity of the whole template chains with query sequence (C) Cov- Represents the coverage of the threading alignment and is equal to the number of aligned residues divided by the length of the query protein. (D) Norm. *Z*-score is the normalized *Z*-score of the threading alignments. Alignment with a normalized *Z*-score >1 means a good alignment and vice versa.

10.7717/peerj.4396/supp-2 Table S2 Consensus prediction of GO terms

10.7717/peerj.4396/supp-3 Table S3 TBP sequences used for phylogeny analysis

10.7717/peerj.4396/supp-4 Figure S1 Conserved proline and glycine residues of pB263R model 1a

10.7717/peerj.4396/supp-5 Figure S2 The predicted structure of the complete pB263R using RaptorX Structure prediction server

10.7717/peerj.4396/supp-6 Data S1 I-TASSER results for the pB263R sequence from amino acids 45–263

10.7717/peerj.4396/supp-7 Data S2 I-TASSER results for the pB263R sequence from amino acids 45–263

10.7717/peerj.4396/supp-8 Data S3 TM-site results of the complete pB263R model

10.7717/peerj.4396/supp-9 Supplemental Information 1 PDB files for the I-TASSER prediction from residue 45–263

10.7717/peerj.4396/supp-10 Supplemental Information 2 COACH Data. COACH Server results for the complete pB263R query sequence

10.7717/peerj.4396/supp-11 Supplemental Information 3 I-TASSER results for the complete pB263R query sequence (Including PDB files)

## Additional Information and Declarations

### Data Availability

The following information was supplied regarding data availability: The raw data is provided in the Supplemental Files.

## References

[ref-1] Afonso CL, Piccone ME, Zaffuto KM, Neilan J, Kutish GF, Lu Z, Balinsky CA, Gibb TR, Bean TJ, Zsak L, Rock DL (2004). African swine fever virus multigene family 360 and 530 genes affect host interferon response. Journal of Virology.

[ref-2] Altschul SF, Madden TL, Schäffer AA, Zhang J, Zhang Z, Miller W, Lipman DJ (1997). Gapped BLAST and PSI-BLAST: a new generation of protein database search programs. Nucleic Acids Research.

[ref-3] Alva V, Nam S-Z, Söding J, Lupas AN (2016). The MPI bioinformatics Toolkit as an integrative platform for advanced protein sequence and structure analysis. Nucleic Acids Research.

[ref-4] Anandapadamanaban M, Andresen C, Helander S, Ohyama Y, Siponen MI, Lundström P, Kokubo T, Ikura M, Moche M, Sunnerhagen M (2013). High-resolution structure of TBP with TAF1 reveals anchoring patterns in transcriptional regulation. Nature Structural & Molecular Biology.

[ref-5] Bateman A, Birney E, Cerruti L, Durbin R, Etwiller L, Eddy SR, Griffiths-jones S, Howe KL, Marshall M, Sonnhammer ELL (2002). The pfam protein families database. Nucleic Acids Research.

[ref-6] Bernstein FC, Koetzle TF, Williams GJ, Meyer EF, Brice MD, Rodgers JR, Kennard O, Shimanouchi T, Tasumi M (1977). The protein data bank: a computer-based archival file for macromolecular structures. Journal of Molecular Biology.

[ref-7] Carrillo C, Borca MV, Afonso CL, Onisk DV, Rock DL (1994). Long-term persistent infection of swine monocytes/macrophages with African swine fever virus. Journal of Virology.

[ref-8] Chapman DAG, Tcherepanov V, Upton C, Dixon LK (2008). Comparison of the genome sequences of non-pathogenic and pathogenic African swine fever virus isolates. The Journal of General Virology.

[ref-9] Colson P, De Lamballerie X, Fournous G, Raoult D (2012). Reclassification of giant viruses composing a fourth domain of life in the new order Megavirales. Intervirology.

[ref-10] Colson P, De Lamballerie X, Yutin N, Asgari S, Bigot Y, Bideshi DK, Cheng X-W, Federici BA, Van Etten JL, Koonin EV, La Scola B, Raoult D (2013). “Megavirales”, a proposed new order for eukaryotic nucleocytoplasmic large DNA viruses. Archives of Virology.

[ref-11] Dimmer E, Huntley R, Barrell D, Binns D, Draghici S, Camon E, Hubank M, Talmud P, Apweiler R, Lovering R (2008). The gene ontology—providing a functional role in proteomic studies. Practical Proteomics.

[ref-12] Dixon LK, Abrams CC, Bowick G, Goatley LC, Kay-Jackson PC, Chapman D, Liverani E, Nix R, Silk R, Zhang F (2004). African swine fever virus proteins involved in evading host defence systems. Veterinary Immunology and Immunopathology.

[ref-13] Dixon LK, Chapman DAG, Netherton CL, Upton C (2013). African swine fever virus replication and genomics. Virus Research.

[ref-14] Donnell VO, Holinka LG, Krug PW, Gladue DP, Carlson J, Sanford B, Alfano M, Kramer E, Lu Z, Arzt J, Reese B, Carrillo C, Risatti GR, Borca V (2015). African swine fever virus georgia 2007 with a deletion of virulence-associated gene 9GL (B119L), when administered at low doses, leads to virus attenuation in swine and induces an effective protection against homologous challenge. Journal of Virology.

[ref-15] Edgar RC, Drive RM, Valley M (2004). MUSCLE: multiple sequence alignment with high accuracy and high throughput. Nucleic Acids Research.

[ref-16] Finn RD, Coggill P, Eberhardt RY, Eddy SR, Mistry J, Mitchell AL, Potter SC, Punta M, Qureshi M, Sangrador-Vegas A, Salazar GA, Tate J, Bateman A (2016). The Pfam protein families database: towards a more sustainable future. Nucleic Acids Research.

[ref-17] Fox NK, Brenner SE, Chandonia J-M (2014). SCOPe: structural classification of Proteins–extended, integrating SCOP and ASTRAL data and classification of new structures. Nucleic Acids Research.

[ref-18] García-Escudero R, Viñuela E (2000). Structure of African swine fever virus late promoters: requirement of a TATA sequence at the initiation region. Journal of Virology.

[ref-19] Huntley RP, Sawford T, Mutowo-Meullenet P, Shypitsyna A, Bonilla C, Martin MJ, O’Donovan C (2015). The GOA database: gene ontology annotation updates for 2015. Nucleic Acids Research.

[ref-20] Iyer LM, Aravind L, Koonin EV (2001). Common origin of four diverse families of large eukaryotic DNA viruses. Journal of Virology.

[ref-21] Juo ZS, Chiu TK, Leiberman PM, Baikalov I, Berk AJ, Dickerson RE (1996). How proteins recognize the TATA box. Journal of Molecular Biology.

[ref-22] Källberg M, Wang H, Wang S, Peng J, Wang Z, Lu H, Xu J (2012). Template-based protein structure modeling using the RaptorX web server. Nature Protocols.

[ref-23] Koster MJE, Snel B, Timmers HTM (2015). Genesis of chromatin and transcription dynamics in the origin of species. Cell.

[ref-24] Lovell SC, Davis IW, Arendall WB, De Bakker PIW, Word JM, Prisant MG, Richardson JS, Richardson DC (2003). Structure validation by C*α* geometry: *ϕ*, *ψ* and C*β* deviation. Proteins: Structure, Function, and Bioinformatics.

[ref-25] Ma J, Wang S, Zhao F, Xu J (2013). Protein threading using context-specific alignment potential. Bioinformatics.

[ref-26] Magrane M, UniProt Consortium (2011). UniProt knowledgebase: a hub of integrated protein data. Database: The Journal of Biological Databases and Curation.

[ref-27] Nikolov DB, Chen H, Halay ED, Usheva AA, Hisatake K, Lee DK, Roeder RG, Burley SK (1995). Crystal structure of a TFIIB-TBP-TATA-element ternary complex. Nature.

[ref-28] Oliveira GP, Andrade AC dos SP, Rodrigues RAL, Arantes TS, Boratto PVM, Silva LKDS, Dornas FP, Trindade G de S, Drumond BP, La Scola B, Kroon EG, Abrahão JS (2017). Promoter motifs in NCLDVs: an evolutionary perspective. Viruses.

[ref-29] Patikoglou GA, Kim JL, Sun L, Yang S-H, Kodadek T, Burley SK (1999). TATA element recognition by the TATA box-binding protein has been conserved throughout evolution. Genes & Development.

[ref-30] Peng J, Xu J (2011). Raptorx: exploiting structure information for protein alignment by statistical inference. Proteins: Structure, Function, and Bioinformatics.

[ref-31] Raush E, Totrov M, Marsden BD, Abagyan R (2009). A new method for publishing three-dimensional content. PLOS ONE.

[ref-32] Reis AL, Abrams CC, Goatley LC, Netherton C, Chapman DG, Sanchez-Cordon P, Dixon LK (2016). Deletion of African swine fever virus interferon inhibitors from the genome of a virulent isolate reduces virulence in domestic pigs and induces a protective response. Vaccine.

[ref-33] Rodríguez JM, Salas ML (2013). African swine fever virus transcription. Virus Research.

[ref-34] Roy A, Kucukural A, Zhang Y (2010). I-TASSER: a unified platform for automated protein structure and function prediction. Nature Protocols.

[ref-35] Roy A, Yang J, Zhang Y (2012). COFACTOR: an accurate comparative algorithm for structure-based protein function annotation. Nucleic Acids Research.

[ref-36] Ruvalcaba-salazar OK, Ram C, Vargas M, Hern R (2005). Recombinant and native Plasmodium falciparum TATA-binding-protein binds to a specific TATA box element in promoter regions. Molecular and Biochemical Parasitology.

[ref-37] Sillitoe I, Lewis TE, Cuff A, Das S, Ashford P, Dawson NL, Furnham N, Laskowski RA, Lee D, Lees JG, Lehtinen S, Studer RA, Thornton J, Orengo CA (2015). CATH: comprehensive structural and functional annotations for genome sequences. Nucleic Acids Research.

[ref-38] Skolnick J, Gao M, Roy A, Srinivasan B, Zhou H (2015). Implications of the small number of distinct ligand binding pockets in proteins for drug discovery, evolution and biochemical function. Bioorganic and Medicinal Chemistry Letters.

[ref-39] Söding J, Biegert A, Lupas AN (2005). The HHpred interactive server for protein homology detection and structure prediction. Nucleic Acids Research.

[ref-40] Tamura K, Stecher G, Peterson D, Filipski A, Kumar S (2013). MEGA6: molecular evolutionary genetics analysis version 6.0. Molecular Biology and Evolution.

[ref-41] Thomas MC, Chiang C-M (2006). The general transcription machinery and general cofactors. Critical Reviews in Biochemistry and Molecular Biology.

[ref-42] Wasmuth EV, Lima CD (2016). UniProt: the universal protein knowledgebase. Nucleic Acids Research.

[ref-43] Waterhouse AM, Procter JB, Martin DMA, Clamp M, Barton GJ (2009). Jalview Version 2—a multiple sequence alignment editor and analysis workbench. Bioinformatics.

[ref-44] Wiederstein M, Sippl MJ (2007). ProSA-web: interactive web service for the recognition of errors in three-dimensional structures of proteins. Nucleic Acids Research.

[ref-45] Wu S, Zhang Y (2007). LOMETS: A local meta-threading-server for protein structure prediction. Nucleic Acids Research.

[ref-46] Xu J, Zhang Y (2010). How significant is a protein structure similarity with TM-score = 0.5?. Bioinformatics.

[ref-47] Yáñez RJ, Rodríguez JM, Nogal ML, Yuste L, Enríquez C, Rodriguez JF, Viñuela E (1995). Analysis of the complete nucleotide sequence of African swine fever virus. Virology.

[ref-48] Yang J, Roy A, Zhang Y (2013a). BioLiP: a semi-manually curated database for biologically relevant ligand-protein interactions. Nucleic Acids Research.

[ref-49] Yang J, Roy A, Zhang Y (2013b). Protein-ligand binding site recognition using complementary binding-specific substructure comparison and sequence profile alignment. Bioinformatics.

[ref-50] Yang J, Yan R, Roy A, Xu D, Poisson J, Zhang Y (2015). The I-TASSER Suite: protein structure and function prediction. Nature Methods.

[ref-51] Yang J, Zhang Y (2015). Protein structure and function prediction using I-TASSER. Current Protocols in Bioinformatics.

[ref-52] Zhang Y (2008). I-TASSER server for protein 3D structure prediction. BMC Bioinformatics.

[ref-53] Zhang Y, Skolnick J (2005). TM-align: a protein structure alignment algorithm based on the TM-score. Nucleic Acids Research.

[ref-54] Zsak L, Caler E, Lu Z, Kutish GF, Neilan JG, Rock DL (1998). A nonessential African swine fever virus gene UK is a significant virulence determinant in domestic swine. Journal of Virology.

