# Supplementary material for: In silico structural and functional prediction of African swine fever virus protein-B263R reveals features of a TATA-binding protein
Source: PeerJ. 2018 Feb 22;6:e4396. doi: 10.7717/peerj.4396 (PMC5825884; doi:10.7717/peerj.4396)
Supplement: Table S1 — (A) Iden1 is the percentage sequence identity of the templates in the threading aligned region with the query sequence. (B) Iden2 is the percentage sequence identity of the whole template chains with query sequence (C) Cov- Represents the coverage of the threading alignment and is equal to the number of aligned residues divided by the length of the query protein. (D) Norm. Z-score is the normalized Z-score of the threading alignments. Alignment with a normalized Z-score >1 means a good alignment and vice versa. [file peerj-06-4396-s001.pdf]

**Table S1.** Top ten analogs identified by LOMETS for threading alignment

| Rank | Protein domain Identity | Organism | PDB ID | Iden1 (%)[a] | Iden2 (%)[b] | Cov[c] | Norm. Z-score[d] |
|---|---|---|---|---|---|---|---|
| 1 | TBP | *Encephalitozoon cuniculi* | 3eikA | 14 | 17 | 0.67 | 4.01 |
| 2 | TBP | *Sulfolobus acidocaldarius* | 1mp9A | 24 | 17 | 0.67 | 1.11 |
| 3 | TBP | *Saccharomyces cerevisiae* | 4b0aA | 16 | 20 | 0.74 | 3.98 |
| 4 | TBP | *Methanocaldococcus jannaschii* | 2z8uA | 16 | 13 | 0.63 | 3.87 |
| 5 | TBP | *Pyrococcus woesei* | 1aisA | 16 | 15 | 0.68 | 3.87 |
| 6 | TBP | *Sulfolobus acidocaldarius* | 1mp9A | 12 | 17 | 0.73 | 3.64 |
| 7 | CPSF-100 | *Saccharomyces cerevisiae* | 2i7xA | 15 | 16 | 0.90 | 0.53 |
| 8 | TBP | *Homo Sapiens* | 1jfiA | 13 | 17 | 0.65 | 0.67 |
| 9 | GS | *Chromohalobacter salexigens* | 5dm3A | 9 | 15 | 0.88 | 0.61 |
| 10 | TBP | *Saccharomyces cerevisiae* | 1ytbA | 27 | 17 | 0.67 | 0.62 |

a) Iden1 is the percentage sequence identity of the templates in the threading aligned region with the query sequence.

b) Iden2 is the percentage sequence identity of the whole template chains with query sequence

c) Cov- Represents the coverage of the threading alignment and is equal to the number of aligned residues divided by the length of the query protein.

d) Norm. Z-score is the normalized Z-score of the threading alignments. Alignment with a normalized Z-score>1 means a good alignment and vice versa

CPSF-100- Cleavage and Polyadenylation Specificity Factor, GS- Glutamate Synthetase, PDB- Protein Data Bank TBP- Tata Binding Protein.
