# Supplementary material for: In silico structural and functional prediction of African swine fever virus protein-B263R reveals features of a TATA-binding protein
Source: PeerJ. 2018 Feb 22;6:e4396. doi: 10.7717/peerj.4396 (PMC5825884; doi:10.7717/peerj.4396)
Supplement: Table S2 [file peerj-06-4396-s002.pdf]

Table S2 Consensus prediction of GO terms

Molecular Function	Go-Score	Biological Process	Go-Score	Cellular Component	Go-Score
GO:0003677-DNA Binding	0.78	GO:0006367-Transcription Initiation from RNA pol II promoter	0.78	GO:0016591 DNA directed RNA Pol II, holoenzyme	0.52
GO:0008135-Translation Factor Activity/Nucleic Acid binding	0.52	GO:0006355-regulation of Transcription, DNA dependent	0.78	GO:0000120 RNA pol I transcription factor complex	0.52
GO:0001071-Nucleic acid binding activity/Transcription factor activity	0.52	GO:0032196 Transposition	0.52	-	-
GO:0003702 RNA pol II transcription activity	0.45	GO:0006384 Transcription Initiation from RNA pol III promoter	0.52	-	-
GO:0005515 Protein binding	0.45	GO:0070897 DNA dependent transcriptional PIC assembly	0.52	-	-
-	-	GO:0044267-Cellular protein metabolic process	0.47	-	-
