# Supplementary material for: In silico structural and functional prediction of African swine fever virus protein-B263R reveals features of a TATA-binding protein
Source: PeerJ. 2018 Feb 22;6:e4396. doi: 10.7717/peerj.4396 (PMC5825884; doi:10.7717/peerj.4396)
Supplement: Table S3 [file peerj-06-4396-s003.pdf]

Table S3. TBP sequences used for phylogeny analysis.

**Tata Binding Proteins**			
	Description	Accession	Organism
**pB263R Isolate**			
pB263R|Ba71v	Uncharacterized protein B263R-Ba71v isolate	Q65175	*African swine fever virus*
**NCDLVs**			
Apm|TBP	TATA-box-binding protein-like	NC_014649.1	*Acanthamoeba polyphaga mimivirus*
Hav| Putative TBP	Putative TATA-box-binding protein	KX008963.1	*Heterosigma akashiwo virus*
Mv|TBP	TATA-box-binding protein-like protein	AEX61565	*Megavirus courdo7*
Ylpv A|Putative TBP	putative TATA box binding protein	YP_009174782	*Yellowstone lake phycodnavirus 1*
Ylpv B|Putative TBP	putative TATA box binding protein	NC_028108.1	*Yellowstone lake phycodnavirus 3*
Pbcv |TFIID/TBP1	transcription factor TFIID (or TATA-binding protein TBP)	AGE48549	*Paramecium bursaria Chlorella virus AN69C*
Pbcv |TFIID/TBP2	transcription factor TFIID (or TATA-binding protein TBP)	AGE48777	*Paramecium bursaria Chlorella virus AP110A*
Atcv |TFIID/TBP	Transcription factor TFIID (Or TATA-binding protein TBP)	AGE56691	*Acanthocystis turfacea Chlorella virus*
**Archaea, Eukaryotes TBP**			
Sc|1ytb_A|TBP	TATA-binding protein	AEP68421	*Saccharomyces cerevisiae*
Sc|4b0a_A|TIF TFIID1	Yeast TATA-binding protein-yeast TAF1	4B0A_A	*Saccharomyces cerevisiae*
Sc|1rm1_A|TFIIA TBP	TATA-binding protein	NP_011075.1	*Saccharomyces cerevisiae*
Sc|TBP	TATA-binding protein	NP_011075.0	*Saccharomyces cerevisiae S288c*
At|TBP2	TATA binding protein 2	NP_175948	*Arabidopsis thaliana*
me| cd04516|TBP	TATA binding protein	OAQ30812	*Mortierella elongata AG-77*
At|TBP1	TATA binding protein 1	NP_187953	*Arabidopsis thaliana*
At|TBP CTD	Chain E, Co-Crystal Structure Of Tbp Recognizing The Minor Groove Of A Tata Element.	1VTL_E	*Arabidopsis thaliana*
At|TBP1	TATA binding protein 1	NP_187953	*Arabidopsis thaliana*
Ec|TBP	Chain C, Crystal Structure Of The Mot1 N-Terminal Domain In Complex With Tbp	3OC3_C	*Encephalitozoon cuniculi*

In|TLF-Iso X1	TATA-box-binding protein-like isoform X1	XP_019186975	*Ipomoea nil*
Ce| TBP-1	TATA-box-binding protein	NP_498635	*Caenorhabditis elegans*
Mmus|TRF3	TATA box-binding protein-like protein 2 isoform 1	NP_951014 XP_140875	*Mus musculus*
Dm|TBP	TATA binding protein	NP_523805	*Drosophila melanogaster*
Dm|TRF	TBP-related factor	NP_476939	*Drosophila melanogaster*
Hs|TBP2	TATA box-binding protein	2119243A	*Homo sapiens*
Hs|TL	TATA box-binding protein-like protein 1	NP_004856	*Homo sapiens*
Hs| 5iyb P|TBP	TATA-box-binding protein isoform 2	NP_001165556	*Homo sapiens*
Hs|TBP	TATA-box-binding protein isoform 1	NP_003185	*Homo sapiens*
mv|Cd00652|TBP TLF	TATA-box-binding protein	KFH70047	*Mortierella verticillata NRRL 6337*
Ce|TLF-1	TBP-Like Factor	NP_492356	*Caenorhabditis elegans*
ag|Cd04517 TLF TBPL	PREDICTED: TATA box-binding protein-like protein 1	XP_018577452	*Anoplophora glabripennis*
Hs|TLP2	TATA box-binding protein-like protein 2	NP_950248	*Homo sapiens*
Dm|TRF-IsoJ	TATA box binding protein-related factor 2, isoform J	NP_511084	*Drosophila melanogaster*
Dm|TRF-IsoH	TATA box binding protein-related factor 2, isoform H	NP_996377	*Drosophila melanogaster*
Sa|1mp9A|TBP TF	TATA sequence-binding protein	Q9UWN7	*Sulfolobus acidocaldarius*
Sa|TBP CTD	TATA-box-binding protein	WP_011278173	*Sulfolobus acidocaldarius*
SsP2|TFIID	TATA box binding protein, hypothetical (tfIID)	AAK41225	*Sulfolobus solfataricus P2*
mk|TF	transcription factor	WP_011019207	*Methanopyrus kandleri AV19*
sp|TBP	TATA-binding protein (TBP)	NP_594566	*Schizosaccharomyces pombe*
Ap|TFIID TBP	transcription factor IID TBP	NP_148219	*Aeropyrum pernix K1*
Tp|TBP	TATA box-binding protein	WP_011751968	*Thermofilum pendens*
Ckc|TFP	Archaeal TATA-box-binding family protein	WP_012310109	*Candidatus Korarchaeum cryptofilum*
Nm|TFP	TATA-box binding family protein	ABX13415	*Nitrosopumilus maritimus SCM1*
Pa|TF	transcription factor TATA-box-binding protein	WP_011008464	*Pyrobaculum aerophilum*
pw|1ais A|TBP	Chain A, Tata-Binding Protein TRANSCRIPTION FACTOR	1AIS_A	*Pyrococcus woesei*
pw|d1aisa2|TBP CTD	TATA-box-binding protein	WP_014734019	*Pyrococcus woesei*
ph|TFP	TATA box binding protein (TBP) Transcription Factor	WP_010885095	*Pyrococcus horikoshii*
pw|d1aisa1| TBP	Chain A, Tata-Binding Protein	1D3U_A	*Pyrococcus woesei*

CTD	TRANSCRIPTION FACTOR		
sa|1mp9_A |TBP TF	Chain A, Tbp From A Mesothermophilic Archaeon,	1MP9_A	*Sulfolobus acidocaldarius*
mj|TBP	TATA-box-binding protein	WP_048202361	*Methanocaldococcus jannaschii*
mk|TF	TATA-box-binding protein-TF	Q8TX38	*methanopyrus kandleri*
ta|TFIID	transcription factor TFIID TBP	WP_010900626	*Thermoplasma acidophilum DSM 1728*
mj|2z8u A|TBP	Chain A TBP	2Z8U_A	*Methanocaldococcus jannaschii*
mj|TFIID	TATA-box-binding protein	Q57930	*Methanocaldococcus jannaschii*
archeal TBP	TATA-box-binding protein	WP_070364144	*Multispecies unclassified Halobacteria*
af|TF	transcription factor	WP_010877880	*Archaeoglobus fulgidus*
hv| TB TIF	TATA-binding transcription initiation factor	WP_004045271	*Haloferax volcanii*
mmaz|TF	Archaeal TATA box binding protein (TBP)	WP_011032977	*Methanosarcina mazei Go1*
mmaz| TF B	Transcription factor TF TBP	WP_011034115	*Methanosarcina mazei Go1*
