# Supplementary figures and images for: In silico structural and functional prediction of African swine fever virus protein-B263R reveals features of a TATA-binding protein
Source: PeerJ. 2018 Feb 22;6:e4396. doi: 10.7717/peerj.4396 (PMC5825884; doi:10.7717/peerj.4396)

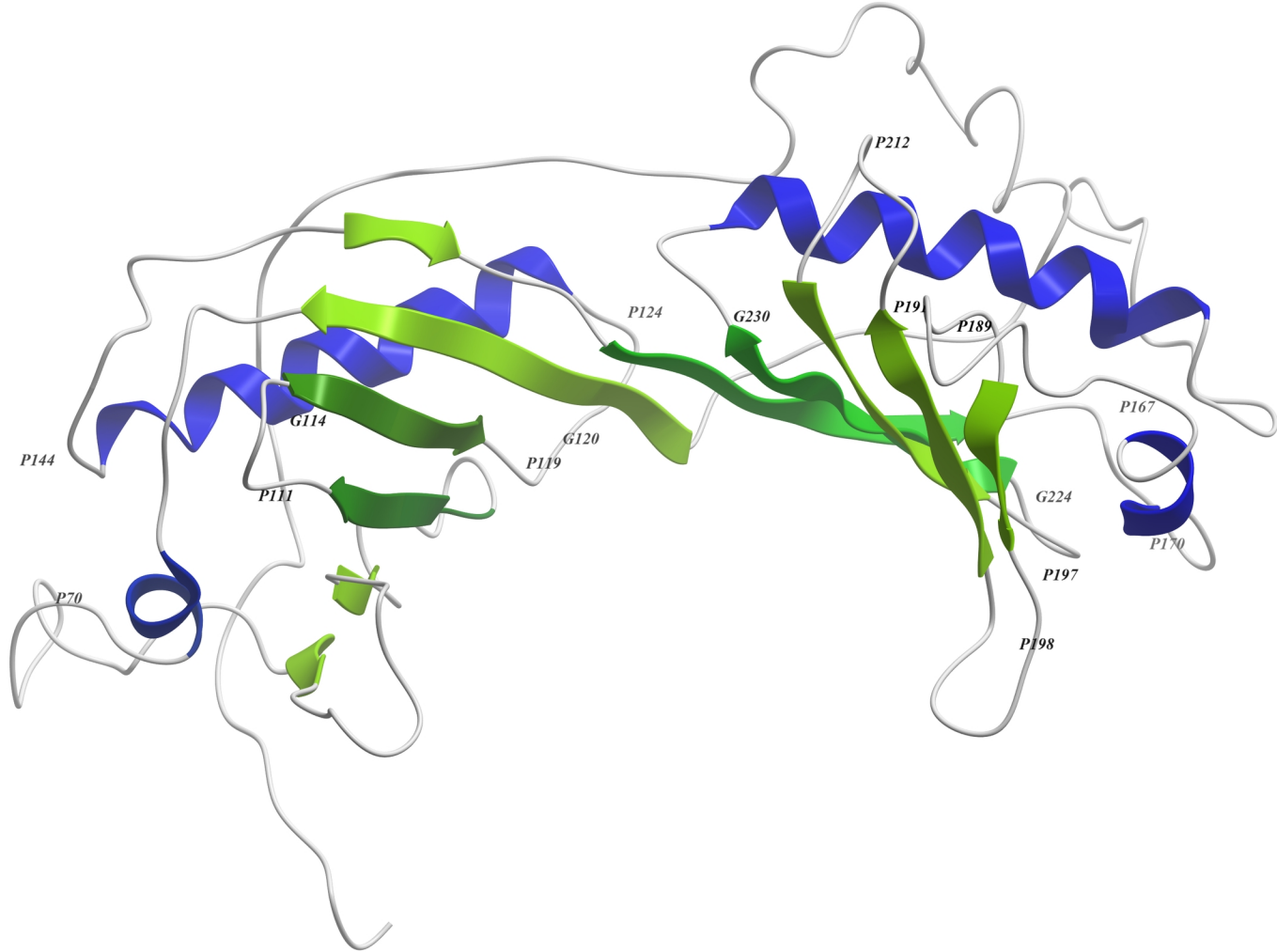

Supplement: Figure S1 [file peerj-06-4396-s004.pdf]

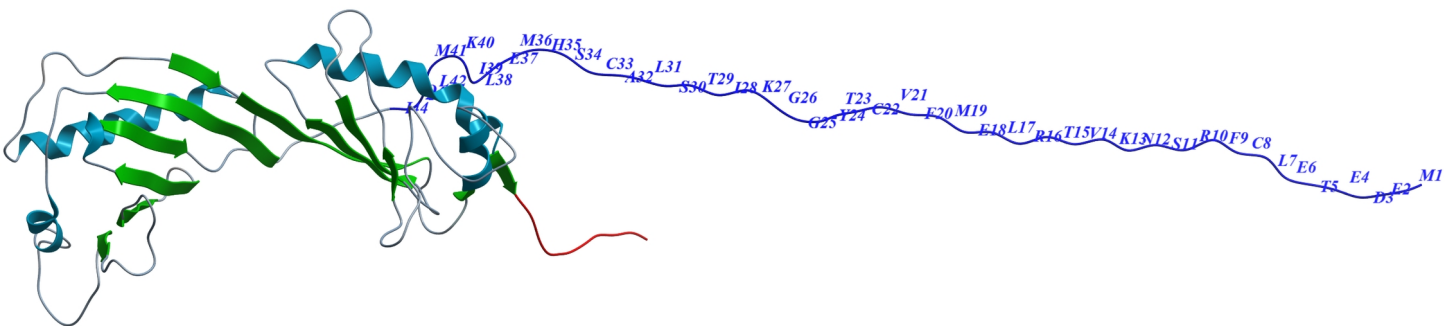

Supplement: Figure S2 [file peerj-06-4396-s005.pdf]

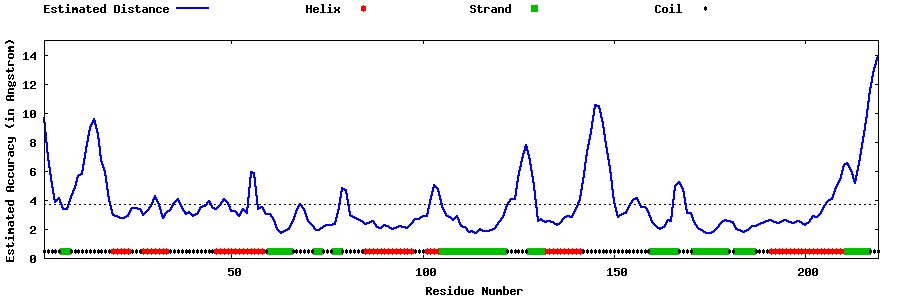

Supplement: Supplemental Information 1 [file peerj-06-4396-s009.bz2 › S313700_results/RSQ_1.png]

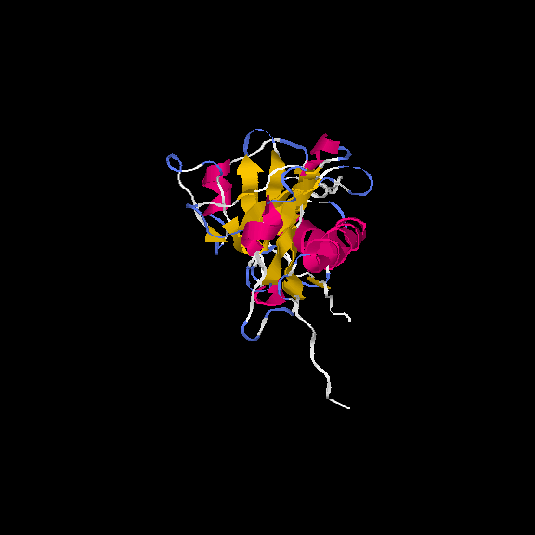

Supplement: Supplemental Information 1 [file peerj-06-4396-s009.bz2 › S313700_results/model1.gif]

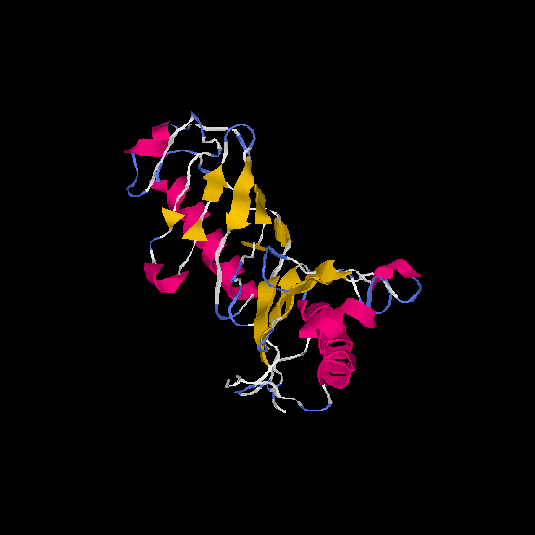

Supplement: Supplemental Information 1 [file peerj-06-4396-s009.bz2 › S313700_results/model2.gif]

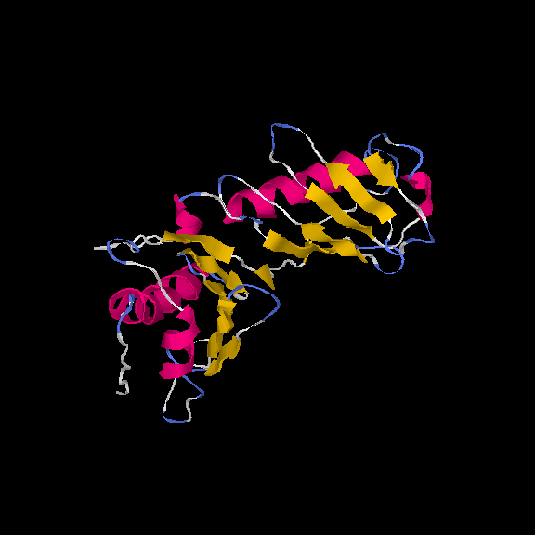

Supplement: Supplemental Information 1 [file peerj-06-4396-s009.bz2 › S313700_results/model5.gif]

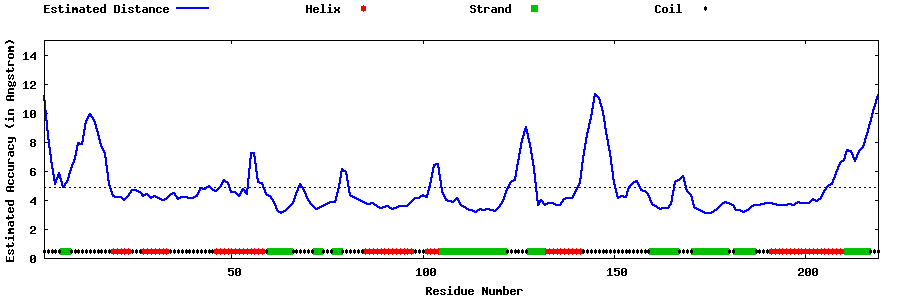

Supplement: Supplemental Information 1 [file peerj-06-4396-s009.bz2 › S313700_results/RSQ_3.png]

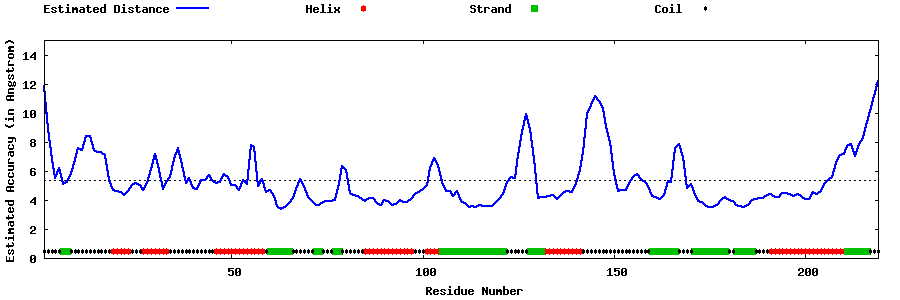

Supplement: Supplemental Information 1 [file peerj-06-4396-s009.bz2 › S313700_results/RSQ_4.png]

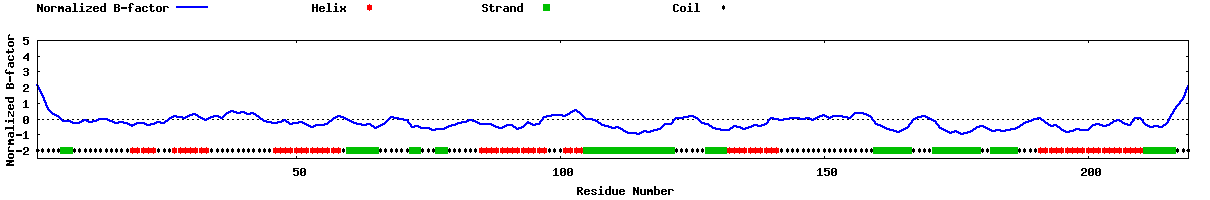

Supplement: Supplemental Information 1 [file peerj-06-4396-s009.bz2 › S313700_results/BFP.png]

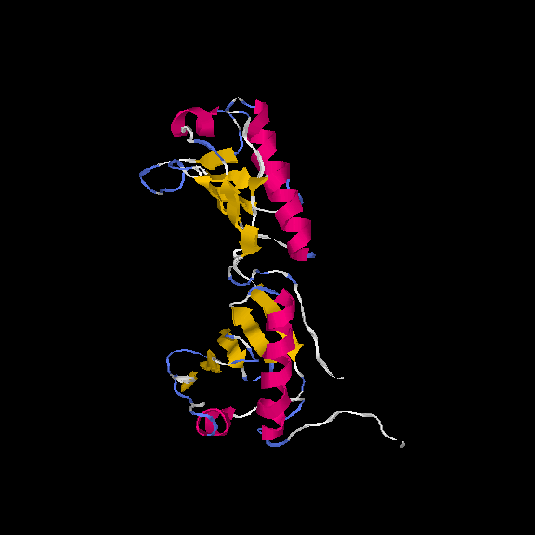

Supplement: Supplemental Information 1 [file peerj-06-4396-s009.bz2 › S313700_results/model4.gif]

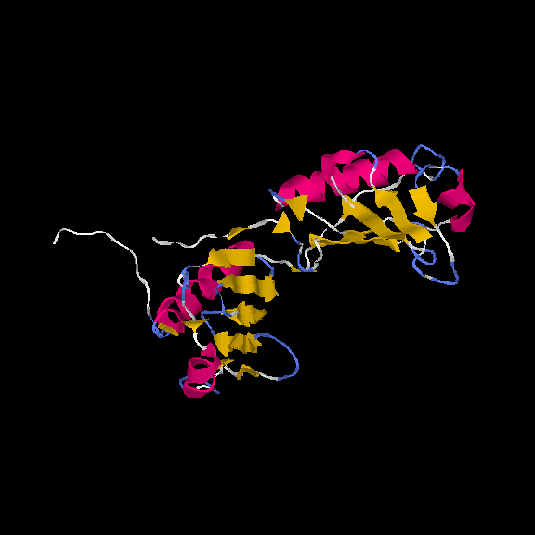

Supplement: Supplemental Information 1 [file peerj-06-4396-s009.bz2 › S313700_results/model3.gif]

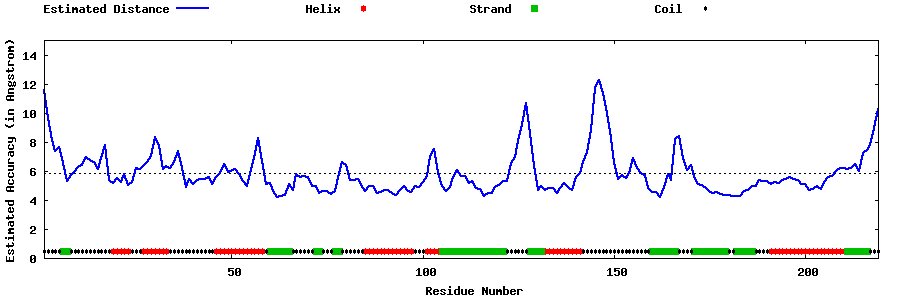

Supplement: Supplemental Information 1 [file peerj-06-4396-s009.bz2 › S313700_results/RSQ_5.png]

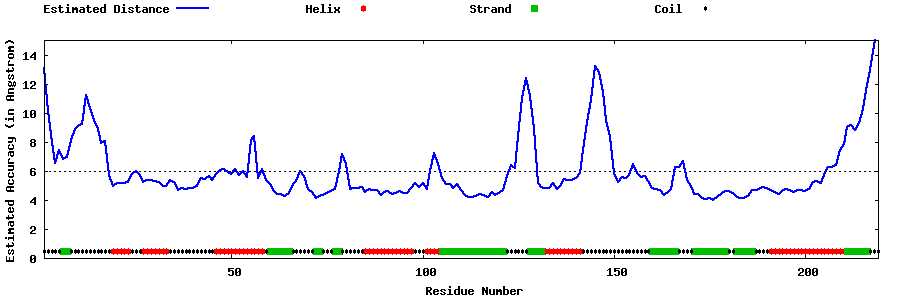

Supplement: Supplemental Information 1 [file peerj-06-4396-s009.bz2 › S313700_results/RSQ_2.png]

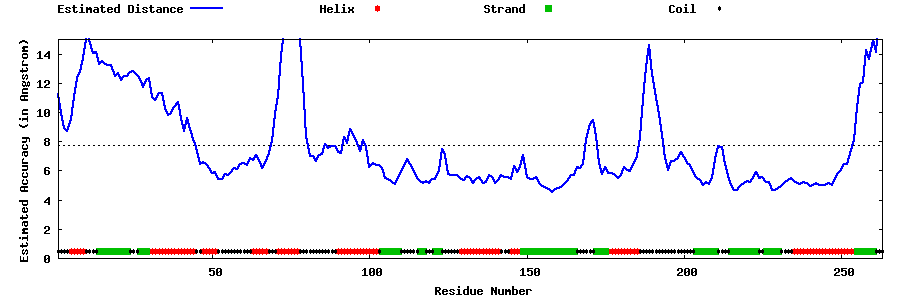

Supplement: Supplemental Information 3 [file peerj-06-4396-s011.bz2 › S281013_results/RSQ_4.png]

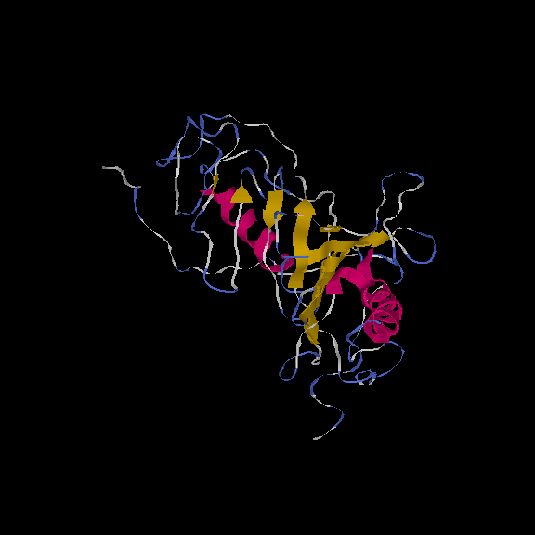

Supplement: Supplemental Information 3 [file peerj-06-4396-s011.bz2 › S281013_results/model5.gif]

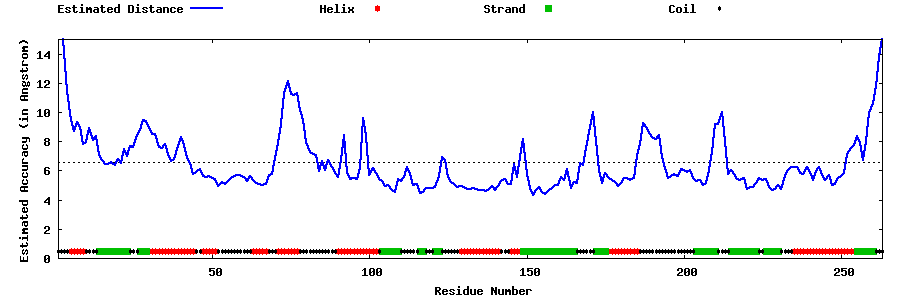

Supplement: Supplemental Information 3 [file peerj-06-4396-s011.bz2 › S281013_results/RSQ_3.png]

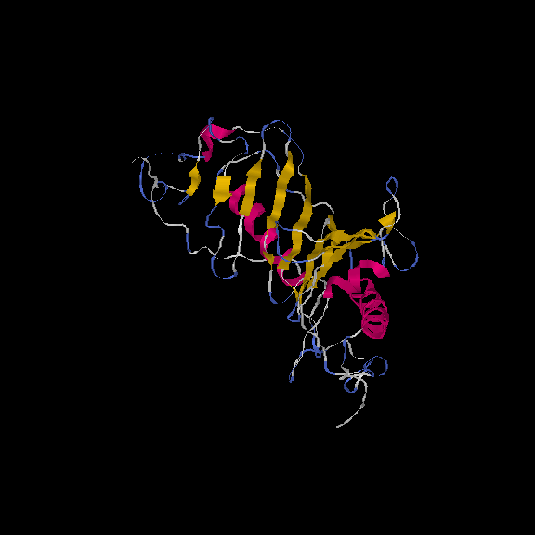

Supplement: Supplemental Information 3 [file peerj-06-4396-s011.bz2 › S281013_results/model2.gif]

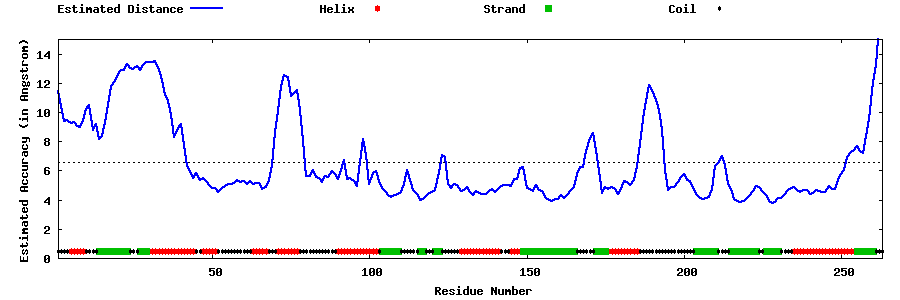

Supplement: Supplemental Information 3 [file peerj-06-4396-s011.bz2 › S281013_results/RSQ_2.png]

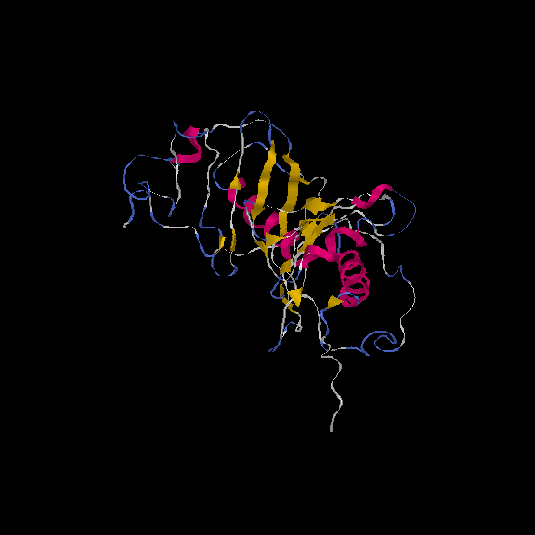

Supplement: Supplemental Information 3 [file peerj-06-4396-s011.bz2 › S281013_results/model3.gif]

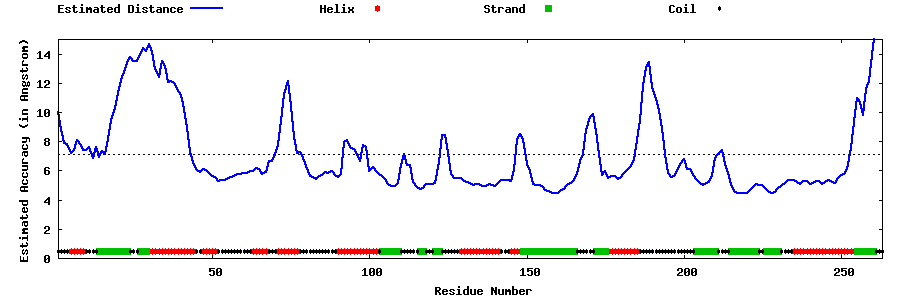

Supplement: Supplemental Information 3 [file peerj-06-4396-s011.bz2 › S281013_results/RSQ_5.png]

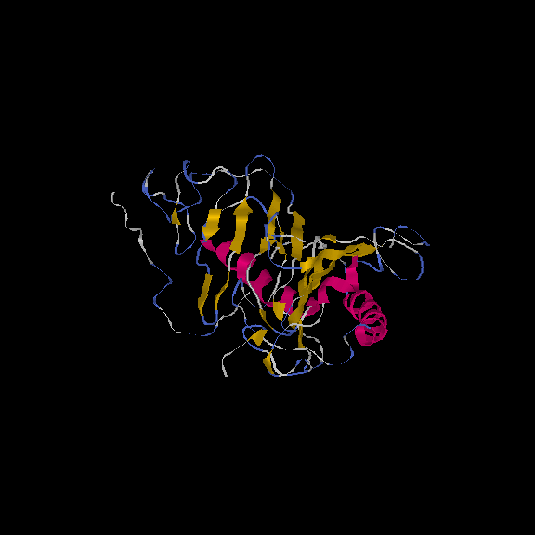

Supplement: Supplemental Information 3 [file peerj-06-4396-s011.bz2 › S281013_results/model4.gif]

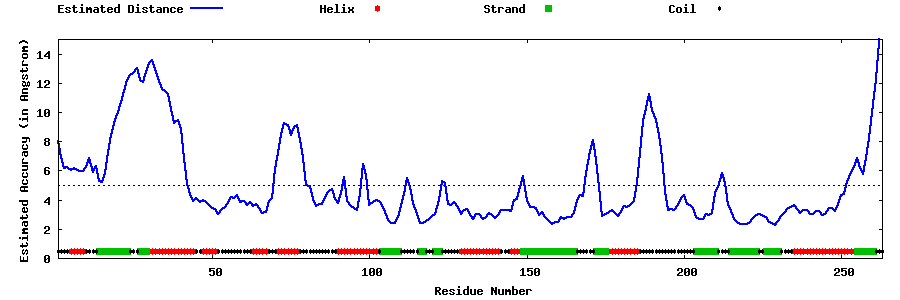

Supplement: Supplemental Information 3 [file peerj-06-4396-s011.bz2 › S281013_results/RSQ_1.png]

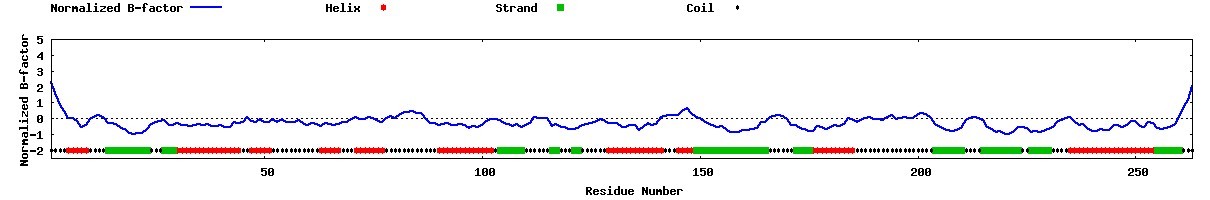

Supplement: Supplemental Information 3 [file peerj-06-4396-s011.bz2 › S281013_results/BFP.png]

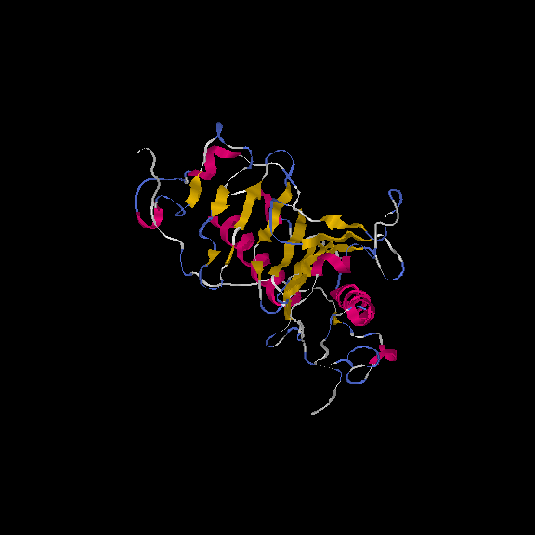

Supplement: Supplemental Information 3 [file peerj-06-4396-s011.bz2 › S281013_results/model1.gif]
