# Supplementary material for: In silico structural and functional prediction of African swine fever virus protein-B263R reveals features of a TATA-binding protein
Source: PeerJ. 2018 Feb 22;6:e4396. doi: 10.7717/peerj.4396 (PMC5825884; doi:10.7717/peerj.4396)
Supplement: Data S1 [file peerj-06-4396-s006.pdf]

S1 Data. I-TASSER results for the complete pB263R query sequence.

[Home] [Server] [Queue] [About] [Remove] [Statistics]

I-TASSER results for job id S281013

[Click on S281013_results.tar.bz2 to download the tarball file including all modeling results listed on this page]

(Click on Annotation of I-TASSER Output to read the instructions for how to interpret the results on this page)

Submitted Sequence in FASTA format

```
>protein
MEDETELCFRSNKVTRLEMFVCTYGGKITSLACSHMELIKMLQIAEPVKALNCNFGHQCL
PGYESLIKTPKKTKNMLRRPRKTEGDGTCFNSAIEASILFKDKMYKLKCFPSTGEIQVPG
VIFPDFEDGKNIIQQWVDFLQHQPIEKKIQIIEFKTIMINFKFQINPVSPRVIIHLKKFA
ALLEHIPTPYPIREIKPPLEDSKVSAKFMVSPGKKVRINVFLKGKINILGCNTKESAEII
YTFLKDLISVHWQEILCVLPVPD
```

Predicted Secondary Structure

```
                     20                  40                  60                  80                  100                 120                 140                 160                 180                 200                 220                 240                 260
                      |                   |                   |                   |                   |                   |                   |                   |                   |                   |                   |                   |                   |
Sequence   MEDETELCFRSNKVTRLEMFVCTYGGKITSLACSHMELIKMLQIAEPVKALNCNFGHQCLPGYESLIKTPKKTKNMLRRPRKTEGDGTCFNSAIEASILFKDKMYKLKCFPSTGEIQVPGVIFPDFEDGKNIIQQWVDFLQHQPIEKKIQIIEFKTIMINFKFQINPVSPRVIIHLKKFAALLEHIPTPYPIREIKPPLEDSKVSAKFMVSPGKKVRINVFLKGKINILGCNTKESAEIIYTFLKDLISVHWQEILCVLPVPD
Prediction CCCCHHHHHCCCCSSSSSSSSSSCCCSSSHHHHHHHHHHHHHHHHCCHHHHHCCCCCCCCCCCHHHHHCCCHHHHHHHCCCCCCCCCCCCHHHHHHHHHHHHHHCSSSSSSCCCCCSSCCCSSCCCCCCHHHHHHHHHHHHCCCHHHHSSSSSSSSSSSSSSSSCCCCCCSSSSHHHHHHHHHHCCCCCCCCCCCCCCCCCCSSSSSSSCCCCSSSSSSSSSCCSSSSSCCCCHHHHHHHHHHHHHHHHHHHHHSSSSSSCCC
Conf.Score 97410102213745789999983286750212217999999875150455358767644756788831936566775187656788542165556554203000246654877714146046777411056999999998854844451479987889998788735889605667888899995189998710148985444110479977996689999971313685216621389999999999887475316961 3799
           H:Helix; S:Strand; C:Coil
```

Predicted Solvent Accessibility

```
                     20                  40                  60                  80                  100                 120                 140                 160                 180                 200                 220                 240                 260
                      |                   |                   |                   |                   |                   |                   |                   |                   |                   |                   |                   |                   |
Sequence   MEDETELCFRSNKVTRLEMFVCTYGGKITSLACSHMELIKMLQIAEPVKALNCNFGHQCLPGYESLIKTPKKTKNMLRRPRKTEGDGTCFNSAIEASILFKDKMYKLKCFPSTGEIQVPGVIFPDFEDGKNIIQQWVDFLQHQPIEKKIQIIEFKTIMINFKFQINPVSPRVIIHLKKFAALLEHIPTPYPIREIKPPLEDSKVSAKFMVSPGKKVRINVFLKGKINILGCNTKESAEIIYTFLKDLISVHWQEILCVLPVPD
Prediction 76452420134531330200001222301310132130031031043042040421240042053004216414521431463634230133013011013332240401143340402000102164034004301410444306430420403201021303043222300010330110053033423055143316654131411133444030201031302011033553040012003200323043000003248
           Values range from 0 (buried residue) to 9 (highly exposed residue)
```

Predicted normalized B-factor

(B-factor is a value to indicate the extent of the inherent thermal mobility of residues/atoms in proteins. In I-TASSER, this value is deduced from threading template proteins from the PDB in combination with the sequence profiles derived from sequence databases. The reported B-factor profile in the figure below corresponds to the normalized B-factor of the target protein, defined by B=(B'-u)/s, where B' is the raw B-factor value, u and s are respectively the mean and standard deviation of the raw B-factors along the sequence. Click here to read more about predicted normalized B-factor)

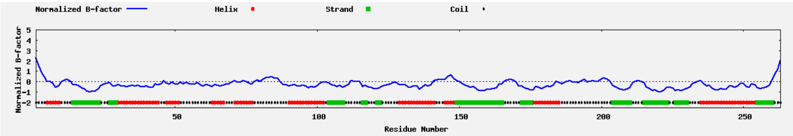

Top 10 threading templates used by I-TASSER

(I-TASSER modeling starts from the structure templates identified by LOMETS from the PDB library. LOMETS is a meta-server threading approach containing multiple threading programs, where each threading program can generate tens of thousands of template alignments. I-TASSER only uses the templates of the highest significance in the threading alignments, the significance of which are measured by the Z-score, i.e. the difference between the raw and average scores in the unit of standard deviation. The templates in this section are the 10 best templates selected from the LOMETS threading programs. Usually, one template of the highest Z-score is selected from each threading program, where the threading programs are sorted by the average performance in the large-scale benchmark test experiments.)

Rank	PDB Hit	Iden1	Iden2	Cov	Norm. Z-score	Download Align.	Alignment
						Sec.Str	CCCCHHHHHCCCCSSSSSSSSSSCCCSSSHHHHHHHHHHHHHHHHCCHHHHHCCCCCCCCCCCHHHHHCCCHHHHHHHCCCCCCCCCCCCHHHHHHHHHHHHHHCSSSSSSCCCCCSSCCCSSCCCCCCHHHHHHHHHHHHCCCHHHHSSSSSSSSSSSSSSSSCCCCCCSSSSHHHHHHHHHHCCCCCCCCCCCCCCCCCCSSSSSSSCCCCSSSSSSSSSCCSSSSSCCCCHHHHHHHHHHHHHHHHHHHHHSSSSSSCCC
						Seq	MEDETELCFRSNKVTRLEMFVCTYGGKITSLACSHMELIKMLQIAEPVKALNCNFGHQCLPGYESLIKTPKKTKNMLRRPRKTEGDGTCFNSAIEASILFKDKMYKLKCFPSTGEIQVPGVIFPDFEDGKNIIQQWVDFLQHQPIEKKIQIIEFKTIMINFKFQINPVSPRVIIHLKKFAALLEHIPTPYPIREIKPPLEDSKVSAKFMVSPGKKVRINVFLKGKINILGCNTKESAEIIYTFLKDLISVHWQEILCVLPVPD
1	3eikA	0.14	0.17	0.67	4.01	Download	---GIIPTLQNVVATVNLSCKLDLKNIALR---------ARNAEYNPKRFA-AVIMR--IREPKTTALIFA-SGKMVITGAK--SEKSSRMAAQRYAKIIHKLGFNA--TFDDFKIQNIVSSCDIKFS-----IRLEGLAYAHSN--------YCSYEELFPGLIYRMV---KPKIVLLIFVSGKIVLTGAKVRDDIYQAFNNIYPVLIQHRK----------
2	1mp9A	0.24	0.17	0.67	1.11	Download	-DEI-----PYKAVVNIENIVATVTL--------------------------DQTLD---------------LYAMERSVPNVEYDPDQFP-----GLIFRLEKITSLIFKS-GKMVVTG--AKSTDELIKAVKRIIKTLKKYGMQPKIQIQNI-VASANLHVIVN---------LDKAAFLLEYEPEQFP-------------GLIYRMDEPR-VVLLIFSSGKMVITGAKRHKAVKKIFDKLVELD--------CVKPVEE
3	4b0aA	0.16	0.20	0.74	3.98	Download	SKDYTEVDFEDEDELAD--------------------------GIVPTLQNIVATVTLGCRLDLKTVALH---------ARNAEYNPKRFA-AVIMR--IREPKTTALIFA-SGKMVVTGAK--SEDDSKLASRKYARIIQKIGFAA--KFTDFKIQNIVGSCDVKFP-----IRLEGLAFSHGT--------FSSYEELFPGLIYRMV---KPKIVLLIFVSGKIVLTGAKQREEIYQAFEAIYPVLSEFRKM---------
4	2z8uA	0.16	0.13	0.63	3.87	Download	--PEIKIVNVVVSTKIGDNIDLEEVAMI---------LENAE--------GLVCR--LSVPKVALLIFR-SGKVNCTGAK--SKEEAEIAIKKIIKELKDAGIDV--IENEIKIQNMVATADLGIE-----PNLDDIALMVEG---------TEYEEQFPGLVYRLD---DPKVVVLIFGSGKVVITGLKSEEDAKRALKKILDTIKEVQ-----------
5	1aisA	0.16	0.15	0.68	3.87	Download	------------------------------------MVD--MSKVKLRIENIVASVDLFAQLDLEKVLDL---------CPNSKYNPEEFP-GIICH--LDDPKVALLIFS-SGKLVVTGAK--SVQDIERAVKLAQKLKSIGVKF---KRPQIDVQNMVFSGDIGRE-----FNLDVVALTLPN---------CEYEEQFPGVIYRVK---EPKSVILLFSSGKIVCSGAKSEADAWEAVRKLLRELDKY------------
6	1mp9A	0.12	0.17	0.73	3.64	Download	-----------------------DEI---------------PYKAVVNIENIVATVTLDQTLDLYAMERS---------VPNVEYDPDQFP-GLIFR--LESPKITSLIFK-SGKMVVTGAK--STDELIKAVKRIIKTLKKYGQLT--GKPKIQIQNIVASANLHVI-----VNLDKAAFLLEN---------NMYEEQFPGLIYRMD---EPRVVLLIFSSGKMVITGAKREDEVHKAVKKIFDKLVELDCVKPVEEEELE
7	2i7xA	0.15	0.16	0.90	0.53	Download	MT--KYNCCDDGSGTTV-GSVVRFDNVTLLIDPSYEQCIKYWEVIPEIDVIILSQPTICLGAHSLLY---YNFTSHFI----SRIQVYATLPVINLGRVSTIDSYASA--GVIGPYDTN---KLDLEDIEISFDHIVPLKYSQLVDLRSRYDGLTLLAYNSIWCISTYSEKLVYNHTRD----NILNAASILDATGKPLSTMRPSAIITTLDRSASIIWPSLKSKIVLSAPKQIQNEEITAKLIKKNIEV------VNMPL-N
8	1jfiA	0.13	0.17	0.65	0.67	Download	----------------QLQNIVSTV------------------------NLGCKL---------DLKTIALRARNAEYNPKRFAA----------VIMRIREPRTTALIF-SSGKMVCTGA--KSEEQSRLAARKYARVVQKLGFPAKFLDFKIQNMVGSCDVKFP-------IRLEGLVLTHQ----------QFSSYEPELFPGLIYRMIKPRIVLLIFVSGKVVLTGAKVRAEIYEAFENIYPILK--------------
9	5dm3A	0.09	0.15	0.88	0.61	Download	ALDDLKTRVESGEIDTVLVCIVDMQGRLMGKRLHARHFVDHGWE----------ETHIMKPDLATLRCVP--WLEGTAMDLLHAEVPHAPRAILKRQLARLEAM-------GLEAIMATELEFFLFEKEEHVLRPLRNHLHAAGI--PVEGTKGEQEELNIR-CAKA--LDTADYHTIAKHATKEIQQGRAVTFLSKWHHAHAGSS-------SHIHQSLWKQGLPAFHDERDALGSALMKHYLAGLLKYAPDYTYFLAPYLN
10	1ytbA	0.27	0.17	0.67	0.62	Download	----------SGIVPTLQNIVATVT-----LGC-RLDLKTVALHAR-----NAEYNPKRFAA---VI--------MIREPKTT-----------ALI-----------FAS-GKMVVTGA--KSEDDSKLASRKYARIIQKIGF--AAKFTDFKIQNIVGSCDVK--FP---IRLEGLAFSHG-TFSSYE-PELFP-----GLIYR-MVKP--KIVLLIFVSGKIVLTGAKQRQAFEAIYPVLSEFRKM------------

(a) All the residues are colored in black; however, those residues in template which are identical to the residue in the query sequence are highlighted in color. Coloring scheme is based on the property of amino acids, where polar are brightly coloured while non-polar residues are colored in dark shade. (more about the colors used)
(b) Rank of templates represents the top ten threading templates used by I-TASSER.
(c) Ident1 is the percentage sequence identity of the templates in the threading aligned region with the query sequence.
(d) Ident2 is the percentage sequence identity of the whole template chains with query sequence.
(e) Cov represents the coverage of the threading alignment and is equal to the number of aligned residues divided by the length of query protein.
(f) Norm. Z-score is the normalized Z-score of the threading alignments. Alignment with a Normalized Z-score >1 mean a good alignment and vice versa.
(g) Download Align. provides the 3D structure of the aligned regions of the threading templates.
(h) The top 10 alignments reported above (in order of their ranking) are from the following threading programs:
1: HHSEARCH2 2: pGenTHREADER 3: HHSEARCH2 4: HHSEARCH2 5: HHSEARCH2 6: HHSEARCH2 7: MUSTER 8: FFAS-3D 9: SPARKS-X 10: SP3

Top 5 final models predicted by I-TASSER

(For each target, I-TASSER simulations generate a large ensemble of structural conformations, called decoys. To select the final models, I-TASSER uses the SPICKER program to cluster all the decoys based on the pair-wise structure similarity, and reports up to five models which corresponds to the five largest structure clusters. The confidence of each model is quantitatively measured by C-score that is calculated based on the significance of threading template alignments and the convergence parameters of the structure assembly simulations. C-score is typically in the range of [-5, 2], where a C-score of a higher value signifies a model with a higher confidence and vice-versa. TM-score and RMSD are estimated based on C-score and protein length following the correlation observed between these qualities. Since the top 5 models are ranked by the cluster size, it is possible that the lower-rank models have a higher C-score in rare cases. Although the first model has a better quality in most cases, it is also possible that the higher-rank models have a better quality than the higher-rank models as seen in our benchmark tests. If the I-TASSER simulations converge, it is possible to have less than 5 clusters generated; this is usually an indication that the models have a good quality because of the converged simulations.)

- More about C-score
- Local structure accuracy profile of the top five models

(By right-click on the images, you can export image file or change the configurations, e.g. modifying the background color or stopping the spin of your models)

- Download Model 1
- C-score=-1.61 (Read more about C-score)
- Estimated TM-score = 0.52±0.15
- Estimated RMSD = 9.5±4.6Å

- Download Model 2
- C-score = -2.51

- Download Model 3
- C-score = -4.09

- Download Model 4
- C-score = -5

- Download Model 5
- C-score = -5.00

Proteins structurally close to the target in the PDB (as identified by TM-align)

(After the structure assembly simulation, I-TASSER uses the TM-align structural alignment program to match the first I-TASSER model to all structures in the PDB library. This section reports the top 10 proteins from the PDB that have the closest structural similarity, i.e. the highest TM-score, to the predicted I-TASSER model. Due to the structural similarity, these proteins often have similar function to the target. However, users are encouraged to use the data in the next section 'Predicted function using COACH' to infer the function of the target protein, since COACH has been extensively trained to derive biological functions from multi-source of sequence and structure features which has on average a higher accuracy than the function annotations derived only from the global structure comparison.)

Top 10 Identified stuctural analogs in PDB

Click to view	Rank	PDB Hit	TM-score	RMSD[a]	IDEN[a]	Cov	Alignment
	1	4b0aA	0.711	1.49	0.162	0.749	Download
	2	1vokA	0.670	1.64	0.135	0.711	Download
	3	1jfiC	0.664	1.45	0.115	0.696	Download
	4	1mp9A	0.657	2.09	0.125	0.711	Download
	5	1nh2A	0.656	1.31	0.167	0.684	Download
	6	3eikA	0.645	1.43	0.146	0.677	Download
	7	1pczA	0.643	1.88	0.158	0.696	Download
	8	2z8uB	0.618	1.68	0.161	0.662	Download
	9	2glsL	0.486	5.48	0.061	0.753	Download
	10	3ng0A	0.481	5.66	0.057	0.760	Download

(a) Query structure is shown in cartoon, while the structural analog is displayed using backbone trace.
(b) Ranking of proteins is based on TM-score of the structural alignment between the query structure and known structures in the PDB library.
(c) RMSD[a] is the RMSD between residues that are structurally aligned by TM-align.
(d) IDEN[a] is the percentage sequence identity in the structurally aligned region.
(e) Cov represents the coverage of the alignment by TM-align and is equal to the number of structurally aligned residues divided by length of the query protein.

Predicted function using COACH

(This section reports biological annotations of the target protein by COACH based on the I-TASSER structure prediction. COACH is a meta-server approach that combines multiple function annotation results from the COFACTOR, TM-SITE and S-SITE programs.)

Ligand binding sites

Click to view	Rank	C-score	Cluster size	PDB Hit	Lig Name	Download Complex	Ligand Binding Site Residues
	1	0.27	34	1qn4A	Nuc.Acid	Rep, Mult	52,54,90,91,108,110,113,115,117,157,158,160,200,201,205,207,217,219,229,232
	2	0.23	29	1qnbA	Nuc.Acid	Rep, Mult	49,50,52,89,90,95,98,104,106,108,119,120,160,201,202,219,221,223,225,227
	3	0.02	2	5iy8P	Nuc.Acid	Rep, Mult	50,97,108,119,202,219,221,223,225,227
	4	0.01	1	2d5nA	ZN	N/A	35,37,53,59
	5	0.01	1	3shyA	MG	N/A	57,102

Download the residue-specific ligand binding probability, which is estimated by SVM.
Download the all possible binding ligands and detailed prediction summary.

Download the templates clustering results.

(a) **C-score** is the confidence score of the prediction. C-score ranges [0-1], where a higher score indicates a more reliable prediction.
(b) **Cluster size** is the total number of templates in a cluster.
(c) **Lig Name** is name of possible binding ligand. Click the name to view its information in the BioLiP database.
(d) **Rep** is a single complex structure with the most representative ligand in the cluster, i.e., the one listed in the **Lig Name** column.
Mult is the complex structures with all potential binding ligands in the cluster.

Enzyme Commission (EC) numbers and active sites

Click to view	Rank	$Cscore^{EC}$	PDB Hit	TM-score	$RMSD^a$	$IDEN^a$	Cov	EC Number	Active Site Residues
	1	0.142	2d3aA	0.462	5.12	0.059	0.688	6.3.1.2	NA
	2	0.141	1lgrA	0.460	5.07	0.039	0.677	6.3.1.2	NA
	3	0.141	2lgsA	0.481	5.45	0.061	0.741	6.3.1.2	NA
	4	0.141	2uu7A	0.459	5.20	0.073	0.696	6.3.1.2	NA
	5	0.141	1htoA	0.477	5.48	0.053	0.738	6.3.1.2	NA

Click on the radio buttons to visualize predicted active site residues.

(a) $Cscore^{EC}$ is the confidence score for the EC number prediction. $Cscore^{EC}$ values range in between [0-1]; where a higher score indicates a more reliable EC number prediction.
(b) TM-score is a measure of global structural similarity between query and template protein.
(c) $RMSD^a$ is the RMSD between residues that are structurally aligned by TM-align.
(d) $IDEN^a$ is the percentage sequence identity in the structurally aligned region.
(e) Cov represents the coverage of global structural alignment and is equal to the number of structurally aligned residues divided by length of the query protein.

Gene Ontology (GO) terms

Top 10 homologous GO templates in PDB

Rank	$Cscore^{GO}$	TM-score	$RMSD^a$	$IDEN^a$	Cov	PDB Hit	Associated GO Terms
1	0.26	0.6471	1.51	0.14	0.68	3eikB	GO:0003700 GO:0006355 GO:0006413 GO:0003743 GO:0003677 GO:0005488 GO:0006367
2	0.26	0.6638	1.45	0.12	0.70	1jfiC	GO:0003677 GO:0005488 GO:0006355 GO:0006367
3	0.26	0.6543	1.33	0.16	0.68	1ngmA	GO:0000126 GO:0000500 GO:0006366 GO:0043565 GO:0005488 GO:0070898 GO:0070893 GO:0005654 GO:0070860 GO:0003682 GO:0006360 GO:0008301 GO:0051123 GO:0003677 GO:0005634 GO:0006383 GO:0005669 GO:0006351 GO:0006355 GO:0006367 GO:0005515 GO:0003702
4	0.26	0.6176	1.68	0.16	0.66	2z8uB	GO:0006355 GO:0003677 GO:0006351 GO:0005488 GO:0006367
5	0.25	0.6696	1.64	0.14	0.71	1vokA	GO:0005515 GO:0006351 GO:0003677 GO:0006355 GO:0005634 GO:0006367 GO:0003702 GO:0005488
6	0.25	0.6429	1.88	0.16	0.70	1pczA	GO:0006367 GO:0003677 GO:0006355 GO:0003702 GO:0005488 GO:0006351
7	0.24	0.6569	2.09	0.12	0.71	1mp9A	GO:0006355 GO:0003677 GO:0006351 GO:0005488 GO:0006367
8	0.14	0.4661	5.04	0.05	0.69	3jpzA	GO:0000166 GO:0016772 GO:0016310 GO:0016301 GO:0005524 GO:0003824 GO:0016740
9	0.14	0.4770	5.48	0.05	0.74	1htoA	GO:0030145 GO:0000287 GO:0009405 GO:0004356 GO:0006542 GO:0005524 GO:0040007 GO:0020012 GO:0000166 GO:0016874 GO:0005886 GO:0051260 GO:0005576 GO:0005525 GO:0005737 GO:0019003 GO:0050897 GO:0005829 GO:0043531 GO:0003824 GO:0006807 GO:0009399
10	0.14	0.4812	5.66	0.06	0.76	3ng0A	GO:0000166 GO:0003824 GO:0004356 GO:0005524 GO:0005737 GO:0006542 GO:0006807 GO:0009399 GO:0016874

Consensus prediction of GO terms

**Molecular Function**	GO:0003677	GO:0008135	GO:0001071	GO:0003702	GO:0005515	
**GO-Score**	0.78	0.52	0.52	0.45	0.45	
**Biological Process**	GO:0006367	GO:0006355	GO:0032196	GO:0006384	GO:0070897	GO:0044267
**GO-Score**	0.78	0.78	0.52	0.52	0.52	0.47
**Cellular Component**	GO:0016591	GO:0000120				
**GO-Score**	0.52	0.52				

(a) $Cscore^{GO}$ is a combined measure for evaluating global and local similarity between query and template protein. It's range is [0-1] and higher values indicate more confident predictions.
(b) TM-score is a measure of global structural similarity between query and template protein.
(c) $RMSD^a$ is the RMSD between residues that are structurally aligned by TM-align.
(d) $IDEN^a$ is the percentage sequence identity in the structurally aligned region.
(e) Cov represents the coverage of global structural alignment and is equal to the number of structurally aligned residues divided by length of the query protein.
(f) The second table shows a consensus GO terms amongst the top scoring templates. The GO-Score associated with each prediction is defined as the average weight of the GO term, where the weights are assigned based on $Cscore^{GO}$ of the template.

[Click on S281013_results.tar.bz2 to download the tarball file including all modeling results listed on this page]

Please cite the following articles when you use the I-TASSER server:

1. J Yang, R Yan, A Roy, D Xu, J Poisson, Y Zhang. The I-TASSER Suite: Protein structure and function prediction. Nature Methods, 12: 7-8, 2015.
2. J Yang, Y Zhang. I-TASSER server: new development for protein structure and function predictions, Nucleic Acids Research, 43: W174-W181, 2015.
3. A Roy, A Kucukural, Y Zhang. I-TASSER: a unified platform for automated protein structure and function prediction. Nature Protocols, 5: 725-738, 2010.
4. Y Zhang. I-TASSER server for protein 3D structure prediction. BMC Bioinformatics, 9: 40, 2008.
