# Supplementary material for: In silico structural and functional prediction of African swine fever virus protein-B263R reveals features of a TATA-binding protein
Source: PeerJ. 2018 Feb 22;6:e4396. doi: 10.7717/peerj.4396 (PMC5825884; doi:10.7717/peerj.4396)
Supplement: Data S3 [file peerj-06-4396-s008.pdf]

S3 Data. TM-site results of the complete pB263R model.

[Back to COACH server]

COACH results for job CH037519

Download the submitted query structure file

COACH Results

Hide

Click to view	Rank	C-score	Cluster size	PDB Hit	Lig Name	Download Complex	Consensus Binding Residues
	1	0.26	32	1qn4A	Nuc.Acid	Rep, Mult	52,54,90,91,108,110,113,115,117,157,158,160,200,201,205,207,217,219,229,232
	2	0.24	29	1qnbA	Nuc.Acid	Rep, Mult	49,50,52,89,90,95,98,104,106,108,119,120,160,201,202,219,221,223,225,227
	3	0.02	2	5iy8P	Nuc.Acid	Rep, Mult	50,97,108,119,202,219,221,223,225,227
	4	0.01	1	1clqA	CA	N/A	52,54,78
	5	0.01	1	3ju5C	MG	N/A	45,73,79
	6	0.01	1	N/A	N/A	N/A	52,117,119
	7	0.01	1	3shyA	MG	N/A	57,102
	8	0.01	2	2b3zA	ZN	N/A	35,53,59
	9	0.01	1	2i0oA	ZN	N/A	150,153
	10	0.01	1	1frvB	F3S	N/A	155,164

Download the residue-specific binding probability, which is estimated by SVM.
Download the predicted bound ligands and detailed prediction summary.
Download the templates clustering results.

(a) **C-score** is the confidence score of the prediction. C-score ranges [0-1], where a higher score indicates a more reliable prediction.
(b) **Cluster size** is the total number of templates in a cluster.
(c) **Lig Name** is the name of possible binding ligand. Click the ligand name to view its information in the BioLiP database.
(d) **Rep** is a single complex structure with the most representative ligand in the cluster, i.e., the one listed in the **Lig Name** column.
Mult is the complex structures with all potential binding ligands in the cluster.

Reset to initial orientation Spin On/Off Reset to initial orientation Spin On/Off

TM-SITE Results

Hide

Click to view	Rank	C-score[a]	Cluster size[b]	Rep Templ[c]	Mult Templ[d]	Ligands[e]	Predicted binding site residues
	1	0.39	32	1gn4A_BS01_NUC	Download	NUC(32)	52,54,90,91,108,110,113,115,117,157,158,160,200,201,205,207,217,219,229,232
	2	0.37	27	1qnbA_BS02_NUC	Download	NUC(27)	49,50,52,89,90,95,98,104,106,108,119,120,160,201,202,219,221,223,225,227
	3	0.19	3	5fmfQ_BS02_NUC	Download	NUC(3)	49,50,88,89,90,91,93,95,97,104,106,108,110,119,120,122,201,202,221,223,225
	4	0.16	1	5fz5O_BS01_NUC	Download	NUC(1)	52,54,60,90,110,112,115,117,158,201,229
	5	0.16	1	5fz5O_BS02_NUC	Download	NUC(1)	49,50,52,89,90,95,99,106,108,119,202

(a) **C-score** is the confidence score of predicted binding site. C-score ranges [0-1], where a higher score indicates a more reliable prediction.

(b) **Cluster size** is the number of templates in a cluster.

(c) **Rep Templ** presents a representaive ligand-protein template from a cluster. The template names are: (PDBIDchain)_(BioLiP site #)_(Ligand ID). Click the corresponding template to download the structure.

(d) **Mult Templ** provides all ligand-protein complex structures in a cluster, where each ligand is separated by "TER".

(e) **Ligands** lists all ligands in a cluster. The numbers in the parentheses are the appearing times of the corresponding ligands. Click the ligand name to visualize its detailed information in BioLiP.

Spin On/Off Spin On/Off

S-SITE Results

Hide

Click to view	Rank	C-score[a]	Cluster size[b]	Templates[c]	Ligands[d]	Predicted binding site residues
	1	0.18	2	List	MG(1),ZN(1)	30,35,53,59,64
	2	0.18	2	List	F3S(1),ZN(1)	35,37,53,59,155,164
	3	0.17	2	List	MG(1),CA(1)	52,54,57,78,102
	4	0.15	1	List	MG(1)	1,3
	5	0.15	1	List	ZN(1)	150,153

(a) **C-score** is the confidence score of predicted binding site. C-score ranges [0-1], where a higher score indicates a more reliable prediction.

(b) **Cluster size** is the number of templates in a cluster.

(c) **Templates** presents the list of templates in a cluster. The template names are: (PDBIDchain)_(BioLiP site #)_(Ligand ID). Click the corresponding template to search the BioLiP database. When the number of templates is >5, click "show all templates" to get the list of all templates in the cluster

(d) **Ligands** lists ligands in a cluster. The numbers in the parentheses are the appearing times for the corresponding ligands. Click the ligand name to visualize its detailed information in BioLiP.

Spin On/Off Spin On/Off

COFACTOR Results

Hide

Click to view	Rank	C-score[a]	PDB Hit	TM-score	RMSD[b]	IDEN[c]	Cov[d]	BS-score[e]	Lig. Name	Download Complex	Predicted binding site residues
	1	0.08	1ytbA	0.655	1.32	0.167	0.684	0.65	Nuc.Acid	Download	89,90,95,97,106,108,110,112,115,117,119,157,158,160,217,219,221,225,227,229,230
	2	0.01	5fmfQ	0.655	1.31	0.167	0.684	0.57	Nuc.Acid	Download	117,157,158,208,219,229,232

(a) **C-score** is the confidence score of predicted binding site. C-score values range in between [0-1]; where a higher score indicates a more reliable prediction.
(b) **RMSD** the RMSD between residues that are structurally aligned by TM-align.
(c) **IDEN** is the percentage sequence identity in the structurally aligned region.
(d) **Cov** represents the coverage of global structural alignment and is equal to the number of structurally aligned residues divided by length of the query protein.
(e) **BS-score** is a measure of local similarity (sequence & structure) between template binding site and predicted binding site in the query structure. Based on large scale benchmarking analysis, we have observed that a BS-score >1 reflects a significant local match between the predicted and template binding site.

Spin On/Off Spin On/Off

FINDSITE Results

Hide

No binding site was predicted by FINDSITE.

ConCavity Results

Hide

Click to view	Rank	C-score	Predicted binding residues
	1	0.14	52,117,119
	2	0.12	201,205,219,227
	3	0.12	63,84,85,86,87,89,90,92,95

Spin On/Off Spin On/Off

Please cite the following articles when you use the COACH server:

1. Jianyi Yang, Ambrish Roy and Yang Zhang, *Protein-ligand binding site recognition using complementary binding-specific substructure comparison and sequence profile alignment*, Bioinformatics, 29: 2588-2595 (2013) (download the PDF file)
2. Jianyi Yang, Ambrish Roy and Yang Zhang, *BioLiP: a semi-manually curated database for biologically relevant ligand-protein interactions.*, Nucleic Acids Research, 41: D1096-D1103 (2013) (download the PDF file).
