# Supplementary material for: In silico structural and functional prediction of African swine fever virus protein-B263R reveals features of a TATA-binding protein
Source: PeerJ. 2018 Feb 22;6:e4396. doi: 10.7717/peerj.4396 (PMC5825884; doi:10.7717/peerj.4396)
Supplement: Supplemental Information 3 [file peerj-06-4396-s011.bz2 › S281013_results/lscore.html]

I-TASSER results


[Back to the Results Page]
  
  

# Top five models with local structure error profiles

The local accuracy is defined as the distance deviation (in Angstrom) between residue positions
in the model and the native structure.
Since the native structure is unknown, the distance errors in the following plots
are estimated by
ResQ
using support vector regression that makes
use of the coverage of threading alignment, divergence of I-TASSER simulation decoys, and
sequence-based secondary structure and solvent accessibility predictions.
The numerical data of the ResQ prediction are listed in this file: lscore.txt.

More details of ResQ and the local structure error prediction can be found at   
J Yang, Y Wang, Y Zhang. ResQ: An approach to unified estimation of B-factor and residue-specific error in protein structure prediction, Journal of Molecular Biology, in press (2015).
